# LIBRA-WA: a web application for ligand binding site detection and protein function recognition

**DOI:** 10.1093/bioinformatics/btx715

**Published:** 2017-11-06

**Authors:** Daniele Toti, Le Viet Hung, Valentina Tortosa, Valentina Brandi, Fabio Polticelli

**Affiliations:** 1 Department of Sciences, University of Roma Tre, Rome, Italy; 2 Department of Science and Technology, Nguyen Tat Thanh University, Ho chi Minh City, Vietnam; 3 National Institute of Nuclear Physics, Roma Tre Section, Rome, Italy

## Abstract

**Summary:**

Recently, LIBRA, a tool for active/ligand binding site prediction, was described. LIBRA’s effectiveness was comparable to similar state-of-the-art tools; however, its scoring scheme, output presentation, dependence on local resources and overall convenience were amenable to improvements. To solve these issues, LIBRA-WA, a web application based on an improved LIBRA engine, has been developed, featuring a novel scoring scheme consistently improving LIBRA’s performance, and a refined algorithm that can identify binding sites hosted at the interface between different subunits. LIBRA-WA also sports additional functionalities like ligand clustering and a completely redesigned interface for an easier analysis of the output. Extensive tests on 373 apoprotein structures indicate that LIBRA-WA is able to identify the biologically relevant ligand/ligand binding site in 357 cases (∼96%), with the correct prediction ranking first in 349 cases (∼98% of the latter, ∼94% of the total). The earlier stand-alone tool has also been updated and dubbed LIBRA+, by integrating LIBRA-WA’s improved engine for cross-compatibility purposes.

**Availability and implementation:**

LIBRA-WA and LIBRA+ are available at: http://www.computationalbiology.it/software.html.

**Supplementary information:**

Supplementary data are available at *Bioinformatics* online.

## 1 Introduction

In recent years, structure-based protein function recognition has gained renewed interest due to the availability of a large number of experimental protein structures, determined within the context of structural genomics initiatives, whose function is unknown (Grabowski *et al.*, 2016; Petrey *et al.*, 2015). In this framework, we recently developed and described LIBRA, a graph theory-based software tool that, given a protein’s structural model, predicts the presence and identity of active sites and/or small molecule ligand binding sites (Viet Hung *et al.*, 2015). Extensive tests carried out on the LigaSite (Dessailly *et al.*, 2008) set of approximately 400 apoproteins indicated that LIBRA was able to identify the correct binding/active site in ∼90% of the cases analyzed, outperforming other structure-based function recognition software such as SiteSeer (Laskowski *et al.*, 2005a,b), EF-Seek (Murakami *et al.*, 2013) and ASSIST (previously developed in our lab; Caprari *et al.*, 2014), while displaying a performance comparable to ProFunc, which employs a combined sequence/structure approach (Laskowski *et al.*, 2005 b). However, the identified correct site ranked first only in 80% of the cases, a suboptimal performance that needed to be improved for LIBRA to be able to handle the most challenging cases. For this purpose, an improved version of LIBRA featuring a novel scoring system has been developed both as a web application, LIBRA-WA and a standalone tool, LIBRA+. The new system also features an improved algorithm to deal with binding sites located at the interface of different protein subunits and clustering of identified ligands according to their chemical similarity. Tests carried out on the same set of apoproteins earlier used on LIBRA demonstrate a significant improvement of the performance, as LIBRA-WA is able to identify the correct binding site in ∼96% of the cases, with the correct site ranking first in ∼94% of the cases. Comparative tests demonstrate that LIBRA-WA has a performance comparable to the state-of-the-art COACH meta-server.

## 2 Materials and methods

### 2.1 LIBRA-WA’s improved engine and features

LIBRA-WA features an improved active/ligand binding site detection engine and a number of additional features, including a redesigned GUI freely accessible online. The core improvement of the engine lies in a novel scoring system, which takes advantage of a clustering process carried out on more than 17 000 unique small molecule ligands stored in the application’s database, based on their SMILES representation. LIBRA-WA, for each alignment record, now provides a score obtained by combining the contributions given by the aligned binding site’s clique size (number of matching residues between the input protein and the target binding site), RMSD value, and the relative size of the cluster containing the ligand. A detailed description of the calculation of this combined score is provided in the Supplementary Material. Besides, the detection algorithm has been further refined by allowing the identification of binding sites hosted at the interface between different subunits. Recognition jobs can be launched against two pre-compiled databases: a ligand binding sites database, including more than 173 000 entries, and a database of active sites derived from the Catalytic Site Atlas (Furnham, 2014) (∼1000 entries) that can be used for the prediction of the catalytic activity of an input protein. For a detailed description of the procedure used to build the two databases, see Viet Hung *et al.* (2015). Aside from that, as a web application, sharing the same architectural framework of (Atzeni *et al.*, 2011a, b; Toti *et al.*, 2012), LIBRA-WA is freely accessible by any web user, who can schedule multiple recognition jobs. Optionally, LIBRA-WA also enables users to create a personal workspace and access their results at a later time, by notifying the users once the jobs’ executions have terminated. Results can be also graphically displayed in three-dimensions via the Jmol HTML5 plug-in (Hanson, 2010). Furthermore, the LIBRA desktop application has been updated by incorporating the new detection engine and the information about the ligand clusters: this new version, which has been dubbed LIBRA+, can read the results exported from LIBRA-WA and is backward-compatible with the output files produced with the original version of LIBRA. A more thorough description of LIBRA-WA’s additional functionalities is reported in the Supplementary Material.

## 3 Results

The effectiveness of LIBRA-WA has been tested on the LigaSite set. A detailed analysis of the results is reported in Supplementary Table S1. As shown in the table, LIBRA-WA finds the biologically relevant ligand/binding site in ∼96% of the cases. More important for the predictive power of the application, the correct ligand/binding site ranks first in ∼94% of all cases. In fact, in ‘real life’ applications, where no functional information is available on the protein of interest, it is essential that the correct prediction is found in the few first-ranking hits. Even removing from the database the holo-proteins present in the LigaSite set, the application still performs fairly well. In fact, LIBRA-WA still identifies a biologically relevant ligand in 88% of the cases, with the correct ligand ranking first in 80% of the cases (Supplementary Table S4). Particularly striking is the ability of LIBRA-WA to pick out similar ligand binding motifs even in structures that do not display significant sequence/structure similarity. One such example, illustrated in Supplementary Figure S3, is that of the *E.coli* adenylate kinase (apoprotein PDB code 4AKE) which, upon ADP binding undergoes a large conformational change (holoprotein PDB code 2ECK). Therefore, the program does not identify the ADP binding site contained in the database entry 2ECK as a correct match. Nonetheless, it correctly identifies the ADP binding site in the input protein by virtue of the structural similarity with the ADP binding site of the human kinesin-8 motor domain (PDB code 3LRE). As detailed in the Supplementary Material, the *E.coli* adenylate kinase and the human kinesin-8 motor domain do not display a similar fold and share a non-significant 11% sequence identity. A combined execution of LIBRA-WA using both the ligand binding sites and the catalytic sites databases allows a user to obtain information on both the location of the binding site, the identity of the ligand(s) and, in case the input protein is an enzyme, its catalytic activity, and thus assign a function to the input protein with high confidence. For example, on the *E.coli* adenylate kinase and using the ligand binding sites database, LIBRA-WA detects as first hit an ADP binding site similar to that of the kinesin-8 motor domain. However, an execution using the catalytic sites database detects as first hit a catalytic site similar to that of *Bacillus stearothermophilus* adenylate kinase (PDB code 1ZIO). Combining the two information together leads to a highly reliable function prediction for the input protein.

## 4 Discussion

In this paper, the development of LIBRA-WA, a web application based on an improved LIBRA engine has been described. By employing an enhanced, composite scoring system, in LIBRA-WA both precision and recall are significantly improved with respect to LIBRA, as it can be clearly seen from the results of the extensive tests detailed in Supplementary Table S1. Furthermore, LIBRA-WA outperforms SiteSeer while displaying a performance comparable to that of COACH (Yang *et al.*, 2013), ranked as the best method in the weekly CAMEO ligand Binding Site Prediction Experiments (Haas *et al.*, 2013), even though the latter uses a combination of structure-based and sequence-based algorithms, while LIBRA-WA is purely structure-based (Supplementary Tables S2 and S3).

*Conflict of Interest*: none declared.

## Supplementary Material

Supplementary Data
