# Supplementary material for: LIBRA-WA: a web application for ligand binding site detection and protein function recognition
Source: Bioinformatics. 2017 Nov 6;34(5):878–80. doi: 10.1093/bioinformatics/btx715 (PMC6192203; doi:10.1093/bioinformatics/btx715)
Supplement: Supplementary Data [file btx715_toti_et_al_supplementary.pdf]

BIOINFORMATICS

Vol. 00 no. 00 2005
Pages 1–31

LIBRA-WA: a web application for ligand binding site detection and protein function recognition

Daniele Toti [1], Le Viet Hung [2], Valentina Tortosa [1], Valentina Brandi [1] and Fabio Polticelli [1,3*]

[1]Department of Sciences, University of Roma Tre, 00146 Rome, Italy
[2]Department of Science and Technology, Nguyen Tat Thanh University, Ho chi Minh City, Vietnam
[3]National Institute of Nuclear Physics, Roma Tre Section, 00146 Rome, Italy

Received on XXXXX; revised on XXXXX; accepted on XXXXX

1 SUPPLEMENTARY METHODOLOGY

1.1 Clusterization of ligands according to their mutual similarity

The ligands stored in LIBRA-WA's binding sites database have been clustered according to their mutual similarity, as computed by an automatic procedure that was meant to compare their SMILES representation. For this purpose, the SMILES information was retrieved from the Protein Data Bank in Europe[0] via a corresponding crawling mechanism, and a similarity threshold (set to 0.7 after a fine-tuning process) was placed for generating the clusters. The actual similarity score was computed by using a combination of Levenshtein and Jaccard distances applied to the SMILES strings. The clusters' semantics is as follows: each ligand has been placed into a cluster so that its similarity score with all of the other members of the cluster is above or equal the given threshold; the ligand with the highest average similarity score with the other members of the cluster has been dubbed the "core" ligand of such a cluster. Each ligand is thus present within one cluster at most. Clusters can be freely browsed by LIBRA-WA users via their corresponding web page, where the ligands making up each cluster are shown.

1.2 Extension and refinements of the alignment output and the scoring mechanism

Results produced by LIBRA-WA have been enriched with respect to LIBRA with additional information and a new scoring mechanism. Specifically, each binding site alignment record displayed within the results of an alignment process now features the information related to the cluster the corresponding ligand falls into (if any), including the core ligand of the cluster, its total size and the number of records within the result page whose ligand falls into that cluster. Alternatively, if the ligand from the record is not included in any cluster, the ligands from other records that are most similar to the considered ligand are shown. Along with the cluster information, a new, refined score mechanism has been implemented to rank more accurately the alignment records. This new score (S_{new}) combines the contributions given by the aligned binding site's clique size (C, corresponding to the number of matching residues between the input protein and the target binding site), RMSD value (R) and the relative size of the cluster containing the ligand (K), each normalized according to the following formulas:

$$C_n = \frac{C - C_{min}}{C_{max} - C_{min}} \quad (1)$$

$$R_n = \frac{1 - (R - R_{min})}{R_{max} - R_{min}} \quad (2)$$

$$K_n = \frac{K - K_{min}}{K_{max} - K_{min}} \quad (3)$$

where min and max indicate the minimum and maximum value of the respective parameter observed in the considered alignment records.

These normalized values are then weighted and linearly combined according to the following formula:

$$S_{new} = w_c * C_n + w_r * R_n + w_k * K_n \quad (4)$$

where w_c = 0.5, w_r = 0.3 and w_k = 0.2. According to its respective value for S_{new}, each alignment record shows a "Confidence" indicator for the quality of the alignment itself, ranging from "green" ($S_{new} >= 0.7$), to "yellow" ($0.4 <= S_{new} < 0.7$), up to "red" ($S_{new} < 0.4$).

1.3 Identification of ligand binding sites across different protein subunits

As a result of the extensive experimentation carried out on LIBRA (Viet Hung *et al.*, 2015), a number of cases were detected where the program could not identify the correct ligand binding site for the input protein, due to the fact that such a site included residues belonging to different protein subunits or "chains". As a matter of fact, the original algorithm was able to identify only binding sites whose residues belonged to the same chain. In order to overcome this weakness of the application, a mechanism has been devised for identifying binding sites across different chains, by considering, for each residue of a given chain, its neighbours from different chains within a distance threshold. Since a naive iterative approach for performing this computation would obviously degrade the efficiency of the algorithm, residues have been thus indexed via a kind of n-dimensional indexes called KD-Trees, by using the three-dimensional coordinates of their geometric center as their index key. Then, the identification of their neighbours was only a matter of computing the respective Euclidean distances between residues belonging to different chains by applying an efficient nearest-neighbour algorithm, as implemented by (Levy *et al.*, 2015). This resulted in an extremely fast computation that leaves the original performance of LIBRA's algorithm virtually unaffected, while increasing its accuracy for such critical cases.

1.4 Additional features and improvements to user experience

Aside from the scientific advancements mentioned in the previous paragraphs, whose purpose was to improve the effectiveness of the alignment process, LIBRA-WA provides the users with an improved user experience and additional features as described below.

1.4.1 User account and personal panel As a web application (sharing the same architectural framework of (Atzeni *et al.*, 2011a,b; Toti *et al.*, 2012)), LIBRA-WA can be freely accessed and used by any web user without

*To whom correspondence should be addressed

[0] http://www.ebi.ac.uk/pdbe-srv/pdbechem/

© Oxford University Press 2005.

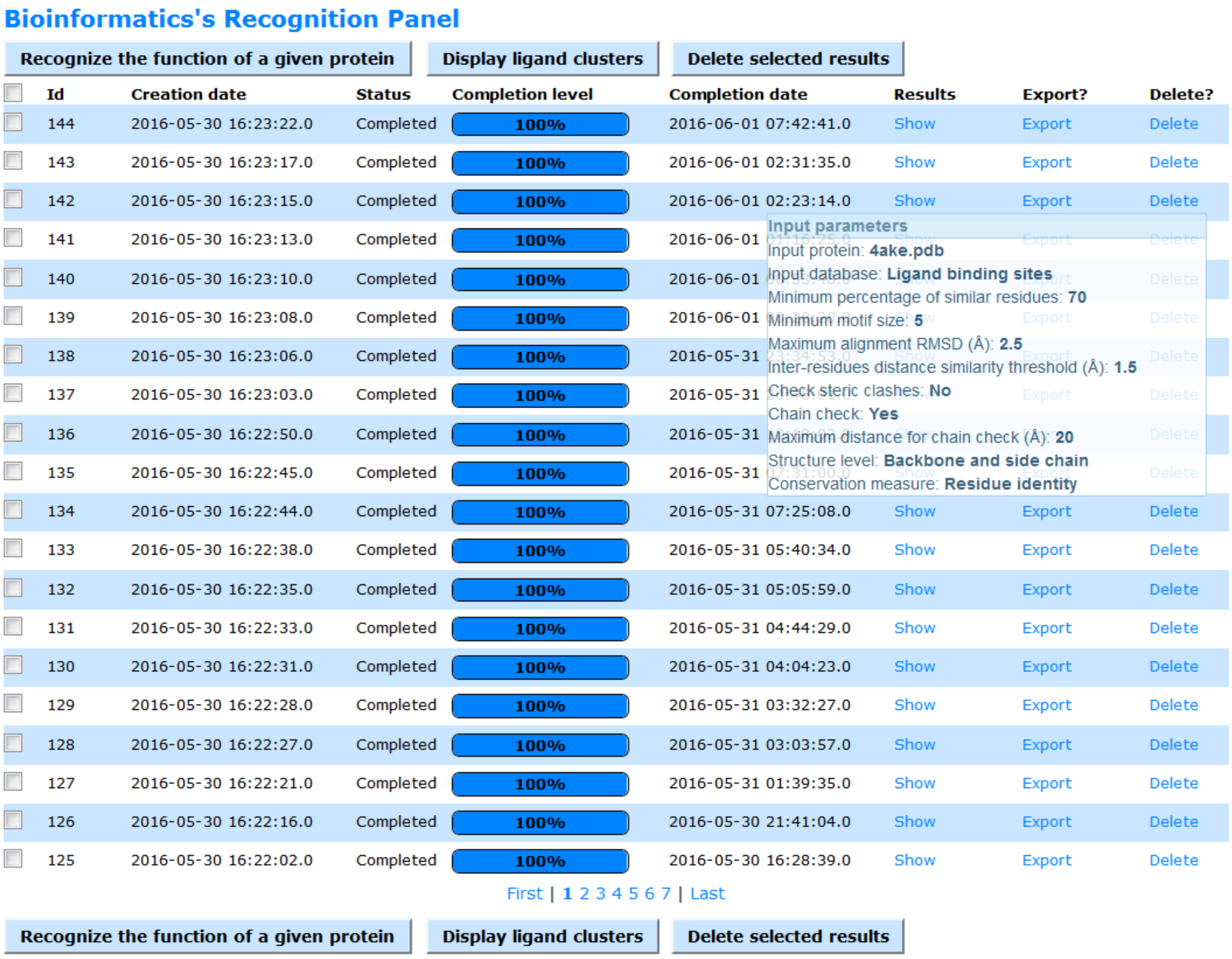

Fig. 1. Screenshot of a user's personal panel in LIBRA-WA, where recognition jobs scheduled, running or completed are shown. For the user's convenience, hovering the mouse over a recognition job displays a summary of the input parameters used when launching it, as shown in the case of the third result from the top. Once available, each result can be browsed in greater detail ("Show"), exported in the LIBRA/LIBRA+ .alg desktop file format ("Export") or deleted altogether ("Delete"). From this panel, a user can access the page where recognitions can be launched ("Recognize the function of a given protein"), as well as browse the ligand clusters generated from the currently available database ("Display ligand clusters").

the need for setting up a personal account; users can start and schedule multiple recognition jobs as they see fit. Optionally, LIBRA-WA allows users to also create a personal workspace on the server by providing a valid e-mail address and selecting a personal password. Once their registration is confirmed via the e-mail sent by the system, they can access their personal panel (as shown in Figure 1). Recognition jobs started by users are automatically placed by the system into its centralized queue and executed in a First-In-First-Out (FIFO) fashion. At the present time, each user can execute only one job at a time while having an unlimited number of scheduled jobs in the queue. Recognition jobs can be launched against a number of pre-compiled databases, including: a manually curated ligand binding sites database, dating back to 2015, with more than 173,000 entries; an unsupervised ligand binding site database, last updated in Q4 2017, including more than 200,000 entries; and a database of active sites derived from the Catalytic Site Atlas (Furnham, 2014) (~1000 entries) that can be used for the prediction of the catalytic activity of an input protein. Registered users can also edit their personal information and log-in credentials via the corresponding page. Each result can be individually analyzed via a corresponding panel showing the alignments produced by the recognition job, as displayed in Figure 2. This panel provides the user with the functionalities to sort the results as needed (by score, clique size, RMSD, protein, ligand, etc.) and filter them by protein, ligand and "core ligand" of a cluster. Besides, the user can display three-dimensional representations of the aligned proteins, check referenced literature for the proteins featured in the database, as well as export the results as described below.

1.4.2 Backward compatibility with LIBRA desktop application and introduction of LIBRA+ The results produced by LIBRA-WA can be easily exported as LIBRA recognition files (.alg) for offline viewing via the desktop application. Additionally, a new version of the desktop application, called LIBRA+, has been released in order to include and display the clusters information and the new scoring mechanism for each of the recognition files: such information is also retroactively available for results produced with the previous version of the desktop application when opened with LIBRA+.

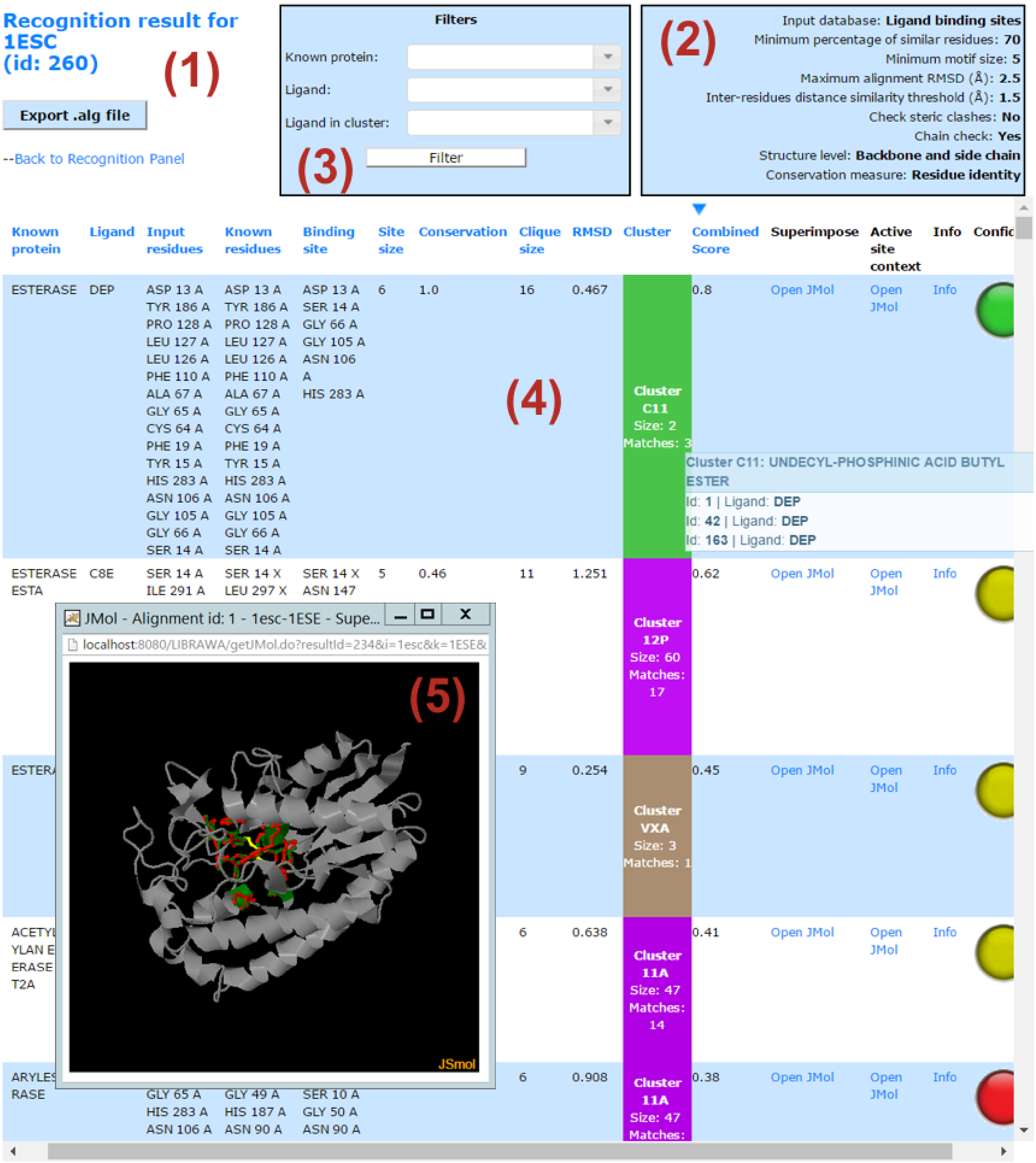

Fig. 2. Screenshot of the panel showing the detailed results for a recognition job. (1) The input protein's PDB code is reported and the possibility of exporting the results in LIBRA+ .alg file format is provided. (2) The values of the input parameters used for the given execution are recapped. (3) Filters are in place for the user's convenience when browsing the results obtained; results can be filtered by known protein, ligand and "core" ligand of a cluster. (4) The results are shown in a table which can be re-ordered as needed by clicking on each column's header. Further information is displayed by hovering the mouse over the known protein and ligand, such as the known protein's PDB ID and ligand's full name, respectively, as well as over the "Cluster" information, where all the records falling into the same clusters are shown (as depicted in the screenshot). For each record, via the Jmol HTML 5 plug-in, three-dimensional representations can be visualized, either showing (i) the input protein with its aligned residues highlighted, upon which the corresponding active site from the known protein is superimposed by means of rototranslations of its residues (as exemplified in the screenshot), or (ii) the known protein where the residues from its binding/active site are highlighted instead. Additional information on the aligned protein from each record, including a reference and PubMed ID of the related literature describing the protein's structure, can be found by clicking on "Info", which opens a corresponding pop-up window. Coloured circles in the right side of results panel represent the "Confidence" indicator as detailed in the text, with green circles characterizing high-confidence results.

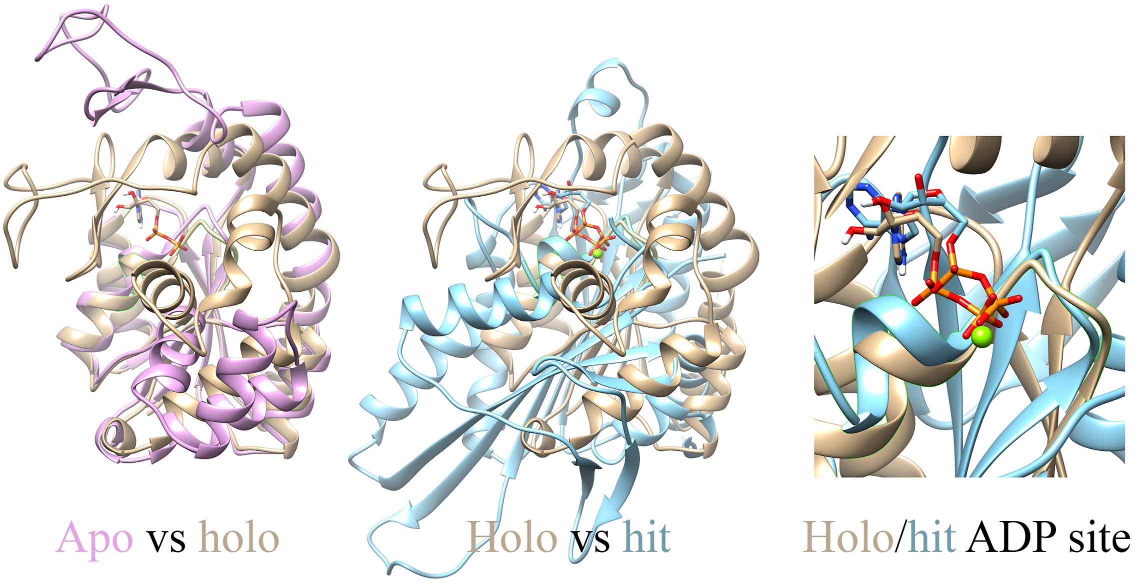

Fig. 3. Left panel. Superimposition of the *E. coli* adenylate kinase in the apo (PDB code 4AKE) and the holo ADP-bound (PDB code 2ECK) form. Note the large conformational change occurring upon ADP binding. Central panel. Superimposition of the ADP-bound structures of the *E. coli* adenylate kinase and the human kinesin-8 motor domain (PDB code 3LRE). Note the lack of structural similarity between the two proteins, paralleled by a lack of significant sequence identity (see text for details). Right panel. Detail of the ADP binding site of the two proteins. Note the good superimposition of the ADP molecules and the local structural similarity of a portion of the α helix and the connected loop hosting the ADP binding site in both proteins (circled in red).

2 SUPPLEMENTARY RESULTS

The effectiveness of LIBRA-WA, and in particular the performance improvement with respect to LIBRA due to the novel scoring mechanism, has been evaluated using the LigaSite set of 373 apo-proteins as a test set. As shown in Table 1 LIBRA-WA finds the biologically relevant ligand/binding site in ~96% of the cases. More importantly, the correct ligand/binding site ranks first in almost all of these cases. In detail, the correct hit is a substrate/substrate analog in 35% of the cases, a specific inhibitor in 16% of the cases, a product/product analog in 13% of the cases, a cofactor/cofactor analog in 13% of the cases, a specific ligand other than the above mentioned categories in 15% of the cases and a transition state analog/reaction intermediate in 3% of the cases. It must be remarked that this performance is in many cases independent from the sequence/structure similarity between the input protein and the holo protein from which the known binding site has been extracted. As already mentioned in the main text, one example of this performance is *E. coli* adenylate kinase (apoprotein PDB code 4AKE) which, upon ADP binding undergoes a large conformational change. As a consequence, LIBRA-WA does not identify the ADP binding site of the corresponding holo protein (PDB code 2ECK) as a correct match. Nonetheless, it correctly identifies the ADP binding site in the input apo protein by virtue of the local, structural similarity with the ADP binding site of the human kinesin-8 motor domain (PDB code 3LRE) which shares a mere 11% sequence identity with the *E. coli* adenylate kinase and practically no significant similarity in the overall protein topology (Figure 3). This observation suggests the possibility to exploit the ligand binding sites database, developed to be used with LIBRA-WA, to analyze if there are common, local structural determinants which govern the binding of specific ligands to different proteins, a possibility which will be explored as one of the future perspectives of this work. Detailed results of the tests performed on the LigaSite set are reported in Table 1. Even removing from the database the holo-proteins present in the LigaSite set, the application still performs fairly well. In fact, LIBRA-WA still identifies a biologically relevant ligand in 88% of the cases, with the correct ligand ranking first in 80% of the cases (Table 4).

For comparative purposes, LIBRA-WA, SiteSeer (Laskowski *et al.*, 2005a,b) and the COACH meta-server (Yang *et al.*, 2013; Roy *et al.*, 2012; Brylinski and Skolnick, 2008; Capra *et al.*, 2009) have been tested on a set of 30 apo-proteins randomly chosen from LigASite. As it can be seen from Table 2, LIBRA-WA finds a correct solution in 97% of the cases (29 out of 30) as opposed to a success rate of 87% for SiteSeer and 90% for COACH meta-server. Particularly interesting is LIBRA-WA's performance in comparison to COACH meta-server as the latter incorporates the results of several different sequence-based and structure-based algorithms (TM-Site, S-Site, COFACTOR, FINDSITE and ConCavity), while LIBRA-WA is, by design, exclusively structure-based; incidentally, COACH meta-server was ranked as the best method in the weekly CAMEO ligand Binding Site Prediction Experiments (Haas *et al.*, 2013). This observation has prompted a more extensive comparison of LIBRA-WA's performance with respect to COACH. For this purpose, COACH's performance has been evaluated on the entire LigaSite test set. The results, shown in Table 3, inidicate that COACH performs slightly better than LIBRA-WA as it finds a biologically relevant ligand in 98% of the cases (versus the 96% success rate of LIBRA-WA). However, the correct result found by COACH ranks first only in 90% of the cases (versus the 94% performance of LIBRA-WA). This is a very good result for LIBRA-WA, given its exclusively structure-based approach and the presence of only small molecules and ions in its database, as opposed to COACH's

database which contains also peptides and nucleic acids. In the light of these extensive tests, it can be stated that LIBRA-WA achieves a performance which is comparable to that of the COACH meta-server.

Table 1: LIBRA-WA's ligand/binding site recognition performance on the LigaSite non-redundant dataset containing 373 apo-proteins for which a corresponding holo structure is available (Desailly *et al.*, 2008). APO and HOLO Protein ID are the PDB codes of the corresponding three-dimensional structures. Ligand ID is the ligand name abbreviation used in the Protein Data Bank.

APO Protein ID	HOLO Protein ID	Ligand ID	Rank	rmsd (Å)	Ligand details
180L	186L	N4B	1st	0.485	Specific ligand analog
1A4U	1B2L	NDC	1st	0.664	Cofactor analog
1ADE	1HOP	GCP	1st	1.023	Substrate analog
1AK1	1C9E	MP1	1st	0.89	Substrate analog
1AKZ	3FCK	FCK	1st	0.593	Specific inhibitor
1ARB	4NSV	2OY	1st	0.324	Specific inhibitor
1ARL	3FVL	BHK	1st	0.533	Specific inhibitor
1B8E	3UEX	STE	1st	0.791	Specific ligand
1B8P	1B8U	NAD	1st	0.558	Cofactor
1BD9	2B7A	OPE	1st	0.648	Natural ligand analog
1BK7	1UCD	U5P	1st	0.456	Reaction product
1BKZ	4UW6	VV7	1st	0.512	Natural ligand
1BQC	2MAN	MAN	1st	0.545	Reaction product
1BYI	1DAG	ACP	1st	0.857	Substrate analog
1C48	1CQF	GAL	1st	0.69	Natural ligand
1C5H	2B46	XYP	1st	0.434	Reaction product
1CEO	1CEN	BGC	1st	0.672	Substrate analog
1CEX	1OXM	TC4	1st	0.484	Covalent inhibitor
1CPJ	2DC7	042	1st	0.654	Covalent inibitor
1CRW	2B4R	NAD	1st	0.729	Cofactor
1CWY	1ESW	ACR	1st	0.543	Specific inhibitor
1DCO	1DCP	HBI	1st	0.713	Cofactor analog
1DHN	2NM3	MPU	1st	0.622	Substrate analog
1DQ0	4P9W	R3M	1st	0.726	Specific ligand analog
1DUP	1DUD	DUD	1st	0.402	Substrate analog
1E4F	4A2B	AGS	1st	0.817	Substrate analog
1E5L	1E5Q	NDP	1st	0.887	Cofactor
1E8Y	3L13	JZW	1st	0.582	Specific inhibitor
1EDQ	1X6N	AO3	1st	0.444	Specific inhibitor
1EPA	1EPB	REA	1st	0.711	Natural ligand
1EWZ	2WGI	PNM	1st	0.936	Specific inhibitor
1EY0	1A2T	THP	1st	0.937	Specific inhibitor
1EZI	1EYR	CDP	1st	0.847	Substrate analog
1F14	1F17	NAI	1st	0.409	Cofactor
1F1S	2BRP	SIE	1st	0.509	Specific inhibitor
1F2V	1I1H	COJ	1st	0.665	Reaction product
1F41	1DVS	STL	1st	0.593	Specific inhibitor
1F5Z	4IMF	SI3	1st	0.723	Substrate
1FCQ	1FCV	NAG	1st	0.7	Substrate fragment
1FGB	1JQY	A32	1st	0.883	Specific inhibitor
1FO9	2APC	UDM	1st	0.863	Substrate analog
1FSF	1HOR	AGP	1st	0.673	Specific inhibitor
1FTF	1FTH	A3P	1st	0.64	Reaction product
1FTR	2FHJ	MFN	1st	0.558	Cofactor
1FWL	1H72	ANP	1st	0.66	Substrate analog
1G40	1RID	IDS	1st	1.783	Natural ligand

1G4E	1G4S	TPS	1st	0.428	Reaction product
1G95	1G97	UD1	1st	0.879	Reaction product
1GBS	1LSP	BUL	1st	0.716	Specific inhibitor
1GCE	1GA0	DVR	1st	0.604	Specific inhibitor
1GFS	1FXS	NAP	1st	0.524	Reaction product
1GOU	1GOY	3GP	1st	1.017	Substrate analog
1GSH	1GSA	GSH	1st	0.773	Reaction product
1GWK	1W8U	BMA	1st	1.254	Natural ligand
1GY0	1OG1	TAD	1st	0.57	Substrate analog
1HK9	3QO3	ATP	1st	0.362	Natural ligand
1HKA	3UDV	J1C	1st	0.592	Substrate analog
1HM5	4QFH	G6P	1st	1.003	Reaction product
1HNK	1HNJ	MLC	1st	0.8	Substrate
1HO1	1HO4	PXP	1st	0.482	Reaction product
1I7N	1I7L	ATP	1st	1.263	Endogenous ligand
1IAD	1QJI	PKF	1st	0.575	Specific inhibitor
1ILV	1J9K	WO4	1st	0.817	Specific inhibitor
1INL	1JQ3	AAT	1st	0.309	Substrate analog
1J85	1MXI	SAH	1st	0.686	Cofactor
1J8S	1J8R	GLA	1st	0.179	Endogenous ligand
1JCF	1JCG	ANP	1st	0.528	Substrate analog
1JKS	1IG1	ANP	1st	0.568	Substrate analog
1JXO	1JXM	MPD	1st	0.434	Non-specific ligand
1JYK	1JYL	CDC	1st	0.701	Reaction product
1K0M	1K0N	GSH	1st	0.87	Substrate
1K3O	4HJ2	LZ6	1st	0.66	Substrate analog
1K6A	1B30	XYP	1st	0.449	Reaction product
1KAM	1KAQ	DND	1st	1.517	Substrate
1KF5	1QHC	PUA	1st	0.802	Reaction product
1KHD	1KGZ	PRP	1st	0.932	Substrate
1KN9	1B12	1PN	1st	0.677	Specific inhibitor
1KPA	1KPE	ADW	1st	0.457	Substrate analog
1KWB	1EIR	BPY	1st	0.406	Substrate
1L7D	1XLT	NAD	1st	1.049	Substrate
1LBV	1LBZ	FBP	1st	0.801	Substrate
1LCI	3RIX	923	1st	0.695	Specific Inhibitor
1LF4	1LF3	EH5	1st	0.541	Specific Inhibitor
1LTU	1LTZ	HBI	1st	0.412	Cofactor
1M1Z	1MB9	ATP	1st	0.753	Substrate
1MKB	4KEH	1r3	1st	0.59	Substrate analog
1MMI	3QSB	743	1st	0.555	Specific inhibitor
1MR7	1KK4	ACO	1st	0.544	Substrate
1MTZ	1MU0	PHK	1st	0.632	Specific inhibitor
1MWK	4A61	ANP	1st	0.985	Substrate analog
1MZL	1FK4	STE	1st	0.697	Natural ligand
1N05	1N07	FMN	1st	1.144	Reaction intermediate
1NDB	2H3P	ACO	1st	0.559	Substrate
1NON	1XZ8	5GP	1st	0.439	Substrate analog
1NXM	2IXL	TRH	1st	0.684	Substrate analog
1O24	1KQ4	FAD	1st	0.501	Cofactor
1OEM	3EAX	LZP	1st	0.827	Specific inhibitor

1OFP	1OF6	DTY	1st	0.906	Specific inhibitor
1OGH	1PKJ	DUT	1st	0.575	Substrate
1OGL	2YB0	DUR	1st	0.82	Substrate analog
1OGM	1OGO	GLC	1st	0.503	Reaction product
1OJQ	1OJZ	NAD	1st	0.877	Cofactor
1OLZ	3OL2	NAG	1st	0.524	Sugar chain
1OOI	2GTE	VA	1st	0.532	Natural ligand
1OPY	3OWY	EQU	1st	0.698	Transition state analog
1OXT	1OXV	ANP	1st	0.534	Reaction product
1P1X	1JCJ	HPD	1st	0.418	Reaction intermediate
1P5H	1T3Z	CAO	1st	0.838	Substrate analog
1P74	3PHJ	DHK	1st	1.024	Reaction product
1PDB	1BOZ	NDP	1st	0.938	Cofactor
1PNG	1PNF	NDG	1st	0.458	Substrate analog
1Q52	1Q51	CAA	1st	0.81	Substrate analog
1Q7M	1Q8J	C2F	1st	0.744	Substrate analog
1QCZ	2FWP	ICR	1st	0.685	Substrate
1QID	1H23	E12	1st	0.322	Specific inhibitor
1QTO	1JIF	BLM	1st	0.703	Natural ligand
1QTR	1X2B	STX	1st	0.415	Specific inhibitor
1R0M	1XPY	NLQ	1st	0.398	Substrate
1R12	3ZWP	AVU	1st	0.601	Substrate analog
1R29	4CP3	RBT	1st	0.761	Specific inhibitor
1RI5	2HV9	SFG	1st	0.301	Specific inhibitor
1RIQ	3HXQ	A5A	1st	0.873	Substrate analog
1RJ1	2CJ7	IOD	1st	0.105	Non-specific ligand
1RKM	2RKM	LYS	4th	1.029	Specific ligand
1RZV	1RZU	ADP	1st	0.655	Reaction product analog
1S2L	1S2D	AR4	1st	0.396	Substrate analog
1S7K	1S7L	COA	1st	0.861	Reaction product
1SGK	1DDT	APU	1st	0.561	Substrate analog
1SGZ	3I25	MV7	1st	0.657	Specific inhibitor
1SJY	1SZ3	GNP	1st	0.121	Product analog
1SNT	1VCU	DAN	1st	0.938	Specific inhibitor
1SQG	1SQF	SAM	1st	0.723	Cofactor
1SUL					**No significant results**
1SWH	3WZP	ZOF	1st	0.715	Specific ligand analog
1TGN	1C2F	BAH	1st	0.887	Specific inhibitor
1THV	4TVT	NA	2nd	0.162	Non-specific ligand
1TIB	1GT6	OLA	1st	0.874	Reaction product
1TJE	1TKY	A3S	1st	0.346	Substrate analog
1TJV	1TJW	AS1	1st	0.834	Substrate analog
1TVN	4A3H	DCB	1st	1.021	Substrate analog
1TW0	1TXC	2AN	1st	0.671	Non-specific ligand
1TYF	2FZS	CMQ	1st	0.508	Specific inhibitor
1TYV	3TH0	PZU	1st	0.632	Natural ligand analog
1TZV	1TZX	CIT	1st	0.987	Specific ligand analog
1U0T	1Y3I	NAD	1st	1.315	Substrate
1U7U	1U7Z	PMT	1st	0.83	Substrate analog
1UAJ	1P9P	SAH	1st	0.701	Substrate analog
1UBI	4WZP	SEP	1st	1.045	Specific ligand
1UF4	1UF5	CDT	1st	0.287	Substrate

1ULU	2WYV	NAD	1st	0.444	Cofactor
1UMF	1UM0	FMN	1st	0.465	Cofactor
1UXZ	1UZ0	BGC	1st	0.337	Specific ligand
1UYL	3T0Z	ATP	1st	0.934	Substrate
1V0S	2ZE9	PD7	1st	0.335	Reaction product
1V6Z	2CX8	SAH	1st	0.626	Cofactor analog
1V77	2CZV	ACY	1st	0.357	Non-specific ligand
1VFJ	1V9O	ADP	1st	0.643	Specific ligand
1VIC	1H7H	CDP	1st	1.172	Substrate analog
1VJU	3DWR	0PA	1st	0.671	Substrate analog
1VPN	1VPS	SIA	1st	0.62	Specific ligand
1W9A	4QVB	F42	1st	0.741	Cofactor
1WJG	2Z08	ATP	1st	0.987	Specific ligand
1WNY	1WNZ	2VA	1st	0.6	Substrate analog
1WOS	1WOP	FFO	1st	0.551	Substrate analog
1WS9	2D29	FAD	1st	0.327	Cofactor
1WTJ	2CWF	NDP	1st	0.457	Cofactor
1WXF	1EE1	ATP	1st	0.556	Substrate analog
1X56	1X55	NSS	1st	0.713	Substrate analog
1X7O	1X7P	SAM	1st	0.534	Cofactor
1X8F	1X6U	DO8	1st	0.457	Reaction product
1XIX	1P4N	UMA	1st	0.225	Substrate
1XK7	1XA4	COA	1st	0.569	Substrate analog
1XO6	1XNY	191	1st	0.548	Substrate
1XQO	1XQP	8HG	1st	0.575	Substrate analog
1XW2	2B4F	XYP	1st	0.381	Reaction product
1Y2Q	3PD2	A3S	1st	0.817	Substrate analog
1Y2T	2OFD	NGA	1st	0.896	Endogenous ligand
1YBT	1YBU	APC	1st	1.21	Substrate analog
1YVY	1YTM	ATP	1st	0.994	Substrate
1Z7G	4RAB	3L3	1st	0.872	Specific inhibitor
1ZAH	3TU9	5MM	1st	0.319	Specific inhibitor
1ZCU	1ZDF	UPG	1st	0.305	Substrate
1ZNW	1ZNX	5GP	1st	0.279	Substrate
1ZTY	1ZU0	CBS	1st	0.815	Natural ligand
1ZUH	3MUF	ADP	1st	1.294	Reaction product
2A6Z					**No significant results**
2A8F	1LRI	CLR	1st	0.897	Specific ligand
2AD1	1BO6	VO4	2nd	0.713	Transition state analog
2AHF	2AHG	UCD	1st	0.617	Substrate
2AHU	2AHV	COA	1st	0.862	Cofactor
2AMJ	2B3D	FAD	1st	0.449	Cofactor
2B0J	3F46	I2C	1st	0.68	Cofactor
2B6P	1YMG	BNG	1st	0.152	Non-specific ligand
2B78	3LDF	SAH	1st	0.405	Cofactor
2B98	2B99	RDL	1st	0.53	Substrate analog inhibitor
2BGT	1J39	UPG	1st	0.96	Substrate
2BJW	2C96	ATP	1st	0.593	Substrate
2BOE	2BOD	BGC	1st	0.267	Reaction product
2C61	3B2Q	ATP	1st	0.592	Reaction product
2C7I	2ARU	ATP	1st	0.555	Substrate
2CAR	2J4E				**No significant results**

2CHS	1COM	PRE	1st	0.599	Reaction product
2CI3	2CI1	KOR	1st	0.654	Specific inhibitor
2CWK	2DY9	ADP	1st	0.678	Substrate
2CX5	2Z0X	5CA	1st	0.717	Substrate analog
2D59	2D5A	COA	1st	0.681	Natural ligand
2DHQ	3N76	CA2	1st	0.515	Specific inhibitor
2DJ6	2DTT	H4B	1st	0.84	Substrate analog
2DKA	2DKC	16G	1st	0.814	Substrate
2DPS	2DPT	PUY	1st	0.532	Substrate analog
2DQW	1EYE	PMM	1st	0.982	Substrate analog
2E0C	2E5M	NAP	1st	0.722	Substrate
2E0K	2E0N	SAH	1st	0.721	Cofactor
2E10	1WQW	BT5	1st	0.409	Substrate analog
2E1V	2E1T	MLC	1st	0.449	Substrate
2E3S	2E3O	16C	1st	0.739	Substrate
2ECR	2ED4	FAD	1st	0.556	Substrate
2EX0	2IIQ	C5P	1st	0.583	Substrate analog
2F82	2FA0	HMG	1st	0.713	Reaction product
2F9T	2F9W	PAU	1st	0.565	Substrate
2FK7	3HA7	B32	1st	0.49	Specific inhibitor
2FP8	2V91	S55	1st	0.691	Substrate
2FSF	2FSH	ANP	1st	0.51	Substrate analog
2G67	2G25	TZK	1st	0.775	Reaction intermediate analog
2G95	3G8E	IS1	1st	0.88	Specific inhibitor
2GFV	2GFX	PMN	1st	0.414	Specific inhibitor
2GG4	1RF6	S3P	1st	1.113	Substrate
2GQV	2P4T	NAP	4th	0.86	Cofactor
2GSF	4P5Z	Q7M	1st	0.924	Specific inhibitor
2GT2	2GT4	GDD	1st	0.623	Substrate
2GUB	4QDW	LAI	1st	0.696	Reaction intermediate
2GWX	3GWX	EPA	1st	0.776	Natural ligand
2GYY	2GZ1	NAP	1st	0.709	Cofactor
2H2Z	2A5K	AZP	1st	0.74	Inhibitor
2HBJ	2HBL	AMP	1st	0.423	Reaction product
2HIV	2HIX	ATP	1st	0.988	Substrate
2HK0	3VNL	TAG	1st	0.357	Substrate
2HVM	1LLO	AMI	1st	0.304	Specific inhibitor
2HY7	2Q6V	UDP	1st	0.966	Substrate analog/reaction product
2HZR	2HZQ	STR	1st	0.589	Natural ligand
2I4L	2I4M	PSD	1st	0.644	Substrate
2IOB	3A2Y	TS5	1st	0.882	Substrate
2IRU	2IRX	GTP	1st	1.127	Substrate
2J71	2J72	GLC	10th	1.18	Natural ligand
2J8N	3DR8	ACO	1st	0.836	Cofactor
2JBR	2JBS	FMN	1st	0.608	Substrate
2NWD	4XAD	3ZW	1st	0.722	Substrate analog
2NXC	3CJT	SAM	1st	0.374	Cofactor
2O9P	2Z1S	CTT	1st	0.403	Substrate
2OAM	2OA1	FAD	1st	0.587	Cofactor
2OPT	3B6A	ZCT	1st	0.959	Specific inhibitor
2OU1	4JGX	PLM	1st	2.203	Natural ligand
2OVE	2OVD	DAO	1st	0.695	Specific ligand

2PAW	4HHY	15R	1st	0.694	Specific inhibitor
2PFK	1PFK	ADP	1st	1.001	Reaction product
2PKF	4O1G	AGS	1st	0.471	Substrate analog
2PPN	1BL4	AP1	1st	0.916	Specific ligand
2Q5R	2JGV	ADP	1st	0.762	Reaction product
2Q6Z	1R3Q	ACP	1st	0.796	Reaction product
2QBV	2W1A	TSA	1st	1.193	Transition state analog
2QDK	1RXU	THM	1st	0.595	Reaction product analog
2QEV	2QEO	LNR	1st	0.412	Natural ligand
2QSU	2QTG	MTH	1st	0.574	Substrate analog
2QVL	2QV7	ADP	1st	0.518	Reaction product
2QYS	2QW8	NAP	1st	0.432	Cofactor
2R60	2R68	SUP	1st	0.24	Reaction product
2RG7	1AIJ	BPH	55th	2.363	Endogenous ligand analog
2RJD	3P8H	P8H	1st	0.248	Specific inhibitor
2SGA	2QAA	TYR	1st	0.559	Reaction intermediate
2SIL	2SIM	DAN	1st	0.179	Specific inhibitor
2TS1	3TS1	TYA	1st	0.41	Reaction intermediate
2UYO	2UYQ	SAM	1st	0.57	Cofactor
2V78	2VAR	ANP	1st	0.494	Substrate
2VFB	2VFC	COA	1st	0.686	Cofactor
2VFY	2VFK	AMP	1st	0.904	Natural ligand
2VQ4	4BFN	GLC	2nd	0.731	Natural ligand
2VUA	2VU9	GAL	1st	0.197	Natural ligand
2WN4	2WN6	NDP	1st	0.768	Cofactor
2WVH	3OE1	TDL	1st	0.457	Reaction intermediate
2WZT	2WZM	NA7	1st	0.928	Cofactor analog
2X5S	2X5Z	GDD	1st	0.829	Reaction product
2YWB	1GPM	AMP	1st	1.03	Product analog
2YXF	1LDS	NA	10th	0.9	Non-specific ligand
2YYA	2YW2	ATP	1st	0.833	Substrate
2YYT	2YYU	C5P	1st	0.481	Product analog
2YZG	2YZN	ANP	1st	0.944	Substrate analog
2ZBS	1S9Q	OHT	1st	1.022	Natural ligand analog
2ZCG	2ZA1	OMP	1st	0.767	Substrate
2ZCO	3ADZ	PS7	1st	0.8	Reaction intermediate
2ZGL	2ZGM	LAT	1st	0.398	Natural ligand analog
2ZHY	2ZHZ	ATP	1st	0.707	Substrate
2ZJ8	2ZJA	ACP	1st	0.717	Substrate analog
2ZTY	3ZZ9	G83	1st	0.645	Specific inhibitor
3A0Y	3A0T	ADP	1st	0.991	Reaction product
3A5Q	1CHW	HXC	1st	0.849	Substrate analog
3AAP	3AAR	ANP	1st	0.793	Specific inhibitor
3ADO	3ADP	NAI	1st	0.632	Cofactor
3APP	1PPM	0P1	1st	0.66	Specific inhibitor
3B3G	2Y1W	SFG	1st	1.05	Cofactor analog
3BA1	3BAZ	NAP	1st	0.583	Cofactor
3BLM	1GHP	PNM	1st	0.481	Reaction intermediate
3BTV	3BTS	NAD	1st	0.827	Specific ligand (activator)
3BUE	3CAG	ARG	1st	0.855	Natural ligand
3BYL	3BYN	RAF	1st	0.762	Substrate analog
3C2E	3C2V	PHT	1st	0.725	Specific inhibitor

3C8N	3B4Y	F42	1st	0.656	Cofactor
3CAF	1E0O	SGN	1st	1.313	Specific ligand
3CB6	3CB5	B3P	30th	0.735	Non-specific ligand
3CGZ	3CGY	RDC	1st	0.849	Specific inhibitor
3COU	2XSQ	IMP	1st	0.497	Reaction product
3CQ1					**No significant results**
3CRM	3CRQ	DPO	1st	0.502	Reaction product
3CSR	3CSZ	NAG	1st	0.249	Substrate analog
3CTB	1ILH	SRL	1st	0.816	Specific agonist
3D0O	3D4P	NAD	1st	0.603	Cofactor
3D95	3CWK	REA	1st	0.683	Endogenous ligand
3DRD	3DOD	PLP	1st	0.954	Cofactor
3DRE	3JU6	ANP	1st	1.295	Substrate analog
3DUL	3DUW	SAH	1st	1.374	Cofactor
3E1S	2EWV	ADP	11th	1.859	Substrate analog
3E5B	3P0X	ICT	1st	0.786	Substrate
3EIZ	3EIY	POP	1st	1.051	Substrate
3EK6	2BND	UDP	1st	1.04	Reaction product
3ETF	3EFV	NAD	1st	0.362	Cofactor
3EX9	3B4P	3B4	1st	0.528	Substrate analog
3EXR	3EXS	5RP	1st	0.574	Product analog
3F1L	3F1K	NAP	1st	0.592	Cofactor
3F6F	3GH6	GSH	1st	0.217	Substrate
3FTD	2ZBR	SFG	1st	1.578	Cofactor analog
3FV6	3FWS	ANP	1st	1.079	Natural ligand analog
3G1S	3G1F	2OM	1st	0.785	Substrate analog
3GBT	3LL3	ATP	1st	0.711	Substrate
3GD0	3GD9	BGC	1st	0.527	Substrate/product analog
3GLK	3GID	S1A	1st	0.483	Specific ligand
3GPG	3GPO	APR	1st	0.58	Specific ligand
3GQH	3GQK	ATP	1st	1.385	Specific ligand
3GSZ	3BR9	DEY	1st	0.547	Specific inhibitor
3H2G	1GKK	SEP	2nd	0.806	Specific ligand
3H38	3H39	ATP	1st	0.883	Substrate
3H49	3IN1	ADP	1st	0.624	Reaction product
3H71	2YA6	DAN	1st	0.604	Specific inhibitor
3HBH	2DKV	MES	1st	0.938	Substrate mimic
3HIS	3HIV	C2X	1st	0.613	Transition state analog
3HJ4	3HIY	UTP	1st	0.874	Substrate
3HNX	3HP8	SUC	1st	0.546	Specific ligand
3I0C	3V0N	3GW	1st	0.831	Specific inhibitor
3I3I	1QLL	TDA	1st	0.688	Specific inhibitor
3I8S	3WIC	GNP	1st	1.196	Substrate analog
3IK8	4TYO	39X	1st	0.419	Specific inhibitor
3ILY					**No significant results**
3ITY	3ITV	PSJ	1st	0.523	Substrate
3IUJ	3IVM	ZPR	1st	0.955	Specific inhibitor
3JYL	3JYN	NDP	1st	0.641	Cofactor
3K0M	4J5A	67Z	1st	0.637	Specific inhibitor
3KAJ	3KAL	HGS	1st	0.633	Reaction product
3KDH	3NJ0	PYV	1st	0.368	Natural ligand mimic

3KJE	3KJG	ADP	1st	1.123	Reaction product
3KJT	4IKM	TYI	1st	0.866	Modified amino acid
3KP7	4EJW	SRY	1st	0.969	Specific inhibitor
3KR9	3KU1	SAM	1st	0.752	Cofactor
3KX7	3KVU	ACO	1st	0.867	Cofactor
3LIG	3LIH	RAF	1st	0.331	Substrate
3LOI	3LZZ	GDP	1st	0.641	Specific ligand
3LXZ	3PR8	GSH	1st	0.209	Substrate
3M4D	3M4E	BCD	1st	0.428	Natural ligand
3N6J	1ECQ	DXG	1st	0.636	Substrate analog
3NK6	3NK7	SAM	1st	0.766	Cofactor
3PTE	1IKG	REX	1st	0.49	Substrate fragment
3SSW	1ZT9	TRP	1st	0.577	Specific ligand
3VZT	3VZU	ANP	1st	0.381	Specific ligand
4AKE	3LR3	ADP	1st	1.273	Reaction product
4PGM	1E59	VO3	1st	1.009	Specific inhibitor
4PTI	1YLC	ABA	1st	1.003	Modified amino acid

Table 2: Comparison of LIBRA-WA, SiteSeer and COACH on their respective ligand/binding site recognition performance, applied on a set of ligand-free proteins randomly chosen from the LigASite. Please note that only the first result provided by each respective system is reported. Wrong results (wrong target protein, ligand not biologically relevant or no results) are highlighted in bold font. Superscript notes for ligands correspond to the following descriptions.1: Substrate analog; 2: Reaction product; 3: Covalent inhibitor; 4: Cofactor; 5: Specific inhibitor; 6: Substrate; 7: Reaction product analog; 8: Natural ligand analog; 9: Cofactor analog; 10: Reaction intermediate; 11: Natural ligand; 12: Non-specific ligand.

**PDB code**	**Experimental function and LigASite ligand**	**Predicted function (PDB code) and ligand (ligand ID)**		
		**LIBRA-WA**	**SiteSeer**	**COACH server**
1AK1	Ferrochelatase<br>N-methylmesoporphyrin	Ferrochelatase (1C9E)<br>N-methylmesoporphyrin (MP1)[1]	Ferrochelatase (1C9E)<br>N-methylmesoporphyrin (MP1)[1]	Ferrochelatase (2HRE)<br>Protoporphyrin IX (PP9)[6]
1BK7	Ribonuclease MC1<br>Uridine-5'-monophosphate	Ribonuclease MC1 (1UCD)<br>Uridine-5'-monophosphate (U5P)[2]	Ribonuclease NW (1IYB)<br>Guanosine-5'-monophosphate (5GP)[2]	Ribonuclease NW (1IYB)<br>Guanosine-5'-monophosphate (5GP)[2]
1CEX	Cutinase<br>Butyl-phosphinic acid 2,3-bis-butylcarbamoyloxy-propyl ester group	Cutinase (1OXM)<br>Butyl-phosphinic acid 2,3-bis-butylcarbamoyloxy-propyl ester (TC4)[3]	Cutinase (1XZM)<br>N-undecanylphosphonate methyl ester group (DPE)[3]	Cutinase (1XZM)<br>N-undecanylphosphonate methyl ester group (DPE)[3]
1CRW	D-Glyceraldheyde-3-phosphate dehydrogenase<br>Nicotinamide-adenine-dinucleotide	Glyceraldehyde-3-phosphate dehydrogenase (2B4R)<br>Nicotinamide-adenine-dinucleotide (NAD)[4]	**D-Glyceraldehyde-3-phosphate dehydrogenase (1DSS)<br>Sulfate ion (SO4)**[12]	Glyceraldehyde-3-phosphate dehydrogenase (4WNC)<br>Nicotinamide-adenine-dinucleotide (NAD)[4]
1EDQ	Chitinase A<br>Allosamizoline	Chitinase A mutant W167A (1X6N)<br>Allosamidin (AO3)[5]	Chitinase A mutant W167A (1X6N)<br>Allosamidin (AO3)[5]	Chitinase 1 (3WL1)<br>N-acetyl-D-glucosamine (NAG)[2]
1EWZ	Beta-Lactamase OXA-10<br>(1R)-2-(1-carboxy-2-hydroxy-2-methyl-propyl)-5,5-dimethyl-thiazolidine-4-carboxylic acid	OXA-10 (2WGI)<br>Open form - penicillin g (PNM)[5]	OXA-10 (1FOF)<br>Cobalt (II) ion (Co)[11]	Beta-lactamase OXA-24 8 (3FZC)<br>(2S,3R)-4-(2-aminoethylcarbamoyloxy)-2-[(2-methanoylindolizin-3-yl)amino]-3-methyl-3-sulfino-butanoic acid (MXF)[5]
1GOU	Ribonuclease<br>Guanosine-3'-monophosphate	Ribonuclease bi (1GOY)<br>Guanosine-3'-monophosphate (3GP)[1]	**Ribonuclease bi (1GOV)<br>Sulfate ion (SO4)**[12]	Ribonuclease bi (1GOY)<br>Guanosine 3'-monophosphate (3GP)[1]
1MR7	Streptogramin A Acetyltransferase<br>Acetyl-coenzyme A	Vat(D) (1KK4)<br>Acetyl coenzyme A (ACO)[6]	Vat(D) (1KK4)<br>Acetyl coenzyme A (ACO)[6]	Hexapeptide-repeat containing-acetyltransferase (3NZ2)<br>Acetyl-coenzyme A (ACO)[6]
1MTZ	Proline iminopeptidase<br>3-amino-1-chloro-4-phenyl-butanol-2-yl	Tricorn interacting factor F1 (1MU0)<br>(2R,3S)-3-amino-1-chloro-4-phenyl-butan-2-ol (PHK)[5]	Tricorn F1-mutant e245q (1XRR)<br>Proline (PRO)[1]	**Gamma lactamase (1HL7)<br>3a,4,7,7a-tetrahydro-benzo [1,3] dioxol-2- one (BD1)**[12]

1NDB	Carnitine acetyltransferase<br>Coenzyme A	Carnitine acetyltransferase (2H3P)<br>Acetyl coenzyme A (ACO)[6]	**No results**	Carnitine acetyltransferase isoform 2 (1S5O)<br>Carnitine (152)[6]
1R0M	N-acylamino acid racemase<br>N-2-acetyl-L-glutamine	N-acylamino acid racemase (1XPY)<br>N 2 -acetyl-L-glutamine (NLQ)[6]	Nacylamino acid racemase (2FKR)<br>N 2 -acetyl-L-glutamine (NLQ)[6]	**Mandelate racemase/muconate lactonizing enzyme family protein (3TOY)**<br>**Calcium ion (Ca)**[12]
1RZV	Glycogen synthase 1<br>Adenosine-5'-diphosphate	Glycogen synthase (1RZU)<br>Adenosine-5'-diphosphate (ADP)[7]	Glycogen synthase (1RZU)<br>Adenosine-5'-diphosphate (ADP)[7]	Glycogen synthase (3GUH)<br>1,5-anhydrosorbitol (ASO)[1]
1SGZ	Beta-secretase<br>Beta-secretase inhibitor	Beta-secretase 1 (3I25)<br>N-[(2S,3S,5R)-1-(3,5-difluorophenoxy)-3-hydroxy-5-(2-methoxyethoxy)-6-[[(2S)-3-methyl-1-oxo-1-(phenylmethylamino)butan-2-yl]amino]-6-oxo-hexan-2-yl]-5-(methyl-methylsulfonyl-amino)-n'-[(1R)-1-phenylethyl]benzene-1,3-dicarboxamide (MV7)[5]	Beta secretase (2OHQ)<br>Glycerol (GOL)[12]	Beta-secretase 1 (2FDP)<br>N1-((2S,3S,5R)-3-amino-6-(4-fluorophenylamino)-5-methyl-6-oxo-1-phenylhexan-2-yl)-n3,n3- dipropylisophthalamide (FRP)[5]
1TIB	Lipase<br>Diundecyl phosphatidyl choline	Lipase (1GT6)<br>Oleic acid (OLA)[2]	Triglyceride lipase (4TGL)<br>Diethyl phosphonate (DEP)[3]	Lipase (1GT6)<br>Oleic acid (OLA)[2]
1TVN	Cellulase<br>Cellobiose	Endoglucanase cel5a (4A3H)<br>2,4-dinitrophenyl-2-deoxy-2-fluoro-beta-D-cellobioside (DCB)[1]	**Endoglucanase cel5g (1TVP)**<br>**4-(2-hydroxyethyl)-1-piperazine ethanesulfonic acid (EPE)**[12]	Endoglucanase D (3NDZ)<br>Cellotriose (CT3)[1]
1V0S	Phospholipase D<br>2-(butyryloxy)-1-[(tetrahydroxyphosphoranyl)oxy] methylethyl butyrate	Phospholipase D (2ZE9)<br>(2r)-3-(phosphonooxy)propane-1,2-diyl diheptanoate (PD7)[2]	Phospholipase D (1U52)<br>1,2-didecanoyl-*sn*-glycero-3-phosphoethanolamine (PEX)[6]	Phospholipase D (1V0R)<br>Tungstate (VI) ion (WO5)[5]
1Y2Q	Threonyl-tRNA synthetase<br>Serine-3'-aminoadenosine	Threonyl-tRNA synthetase (3PD2)<br>Serine-3'-aminoadenosine (A3S)[1]	Threonyl-tRNA synthetase (2HL0)<br>Serine-3'-aminoadenosine (A3S)[1]	Threonyl-tRNA synthetase (4RR6)<br>Serine-3'-aminoadenosine (A3S)[1]
1ZUH	Shikimate kinase<br>Shikimate-3-phosphate	Shikimate kinase (3MUF)<br>Adenosine-5'-diphosphate (ADP)[2]	Shikimate kinase (1ZUI)<br>Trihydroxycyclohex-1-ene-1-carboxylic acid (SKM)[6]	**APS kinase domain of the PAPS synthetase 1 (2OFW)**<br>**Adenosine-5'-phosphosulfate (ADX)**[6]
2CHS	Chorismate mutase<br>Prephenic acid	Chorismate mutase (1COM)<br>Prephenic acid (PRE)[2]	Chorismate mutase (1COM)<br>Prephenic acid (PRE)[2]	Chorismate mutase (1COM)<br>Prephenic acid (PRE)[2]
2DPS	Leucyl/phenylalanil-tRNA-protein transferase<br>Puromycin	Leucyl/phenylalanyl-tRNA-protein transferase (2DPT)<br>Puromycin (PUY)[1]	Leucyl/phenylalanyl-tRNA-protein transferase (2DPT)<br>Puromycin (PUY)[1]	Leucyl/phenylalanyl-tRNA-protein transferase (2Z3O)<br>Phenylalanine (PHE)[6]
2GT2	GDP-mannose mannosyl hydrolase<br>Guanosine-5'-diphosphate	GDP-mannose mannosyl hydrolase (2GT4)<br>Guanosine-5'-diphosphate-alpha-d-mannose (GDD)[6]	GDP-mannose mannosyl hydrolase (2I8T)<br>Guanosine-5'-diphosphate-alpha-d-mannose (GDD)[6]	GDP-mannose mannosyl hydrolase (2GT4)<br>Guanosine-5'-diphosphate-alpha-d-mannose (GDD)[6]
2O9P	Beta-glucosidase B<br>Cellotetraose	Beta-glucosidase B (2Z1S)<br>Beta-D-glucopyranosyl-(1->4)-beta-D-glucopyranosyl-(1->4)-beta-D-glucopyranosyl-(1->4)-beta-D-glucopyranose (CTT)[6]	Beta-glucosidase B (2JIE)<br>2-deoxy-2-fluoro-alpha-D-glucopyranose (G2F)[7]	Beta-glucosidase A (1OIM)<br>Moranoline (NOJ)[5]
2SIL	Sialidase<br>2-deoxy-2,3-dehydro-N-acetyl-neuraminic acid	Neuraminidase (2SIM)<br>2-deoxy-2,3-dehydro-N-acetyl-neuraminic acid (DAN)[5]	Neuraminidase (1SIL)<br>2-deoxy-2,3-dehydro-N-acetyl-neuraminic acid (DAN)[5]	Trans-sialidase (2AH2)<br>3-fluorosialic acid (FSI)[5]
2V78	Fruktokinase<br>Adenosine monophosphate	2-keto-3-deoxygluconate kinase (2VAR)<br>Phosphoaminophosphonic acid-adenylate ester (ANP)[6]	2-keto-3-deoxygluconate kinase (2DCN)<br>Trihydroxy-5-[(phosphonatooxy)methyl] tetrahydrofuran-2-carboxylic acid (CKP)[7]	Ketohexokinase (3QAI)<br>N 8 -(cyclopropylmethyl)-2-(2,6-diazaspiro[3.3]hept- 2-yl)-n 4 -[2-(methylsulfanyl)phenyl] pyrimido[5,4- d]pyrimidine-4,8-diamine (XNN)[5]
2VQ4	Glucoamylase A<br>Glucose	**Acyl carrier protein (3EJB)**<br>**Heptyl 1-thiohexopyranoside (HTG)**[8]	**No results**	Glucoamylase A (2V8L)<br>Alpha-D-glucose (GLC)[11]

2WZT	Aldo-ketoreductase (2R,3R,4R,5R)-5-(6-amino-9H-purin-9-yl)- 3-hydroxy-4-(phosphonooxy)-tetrahydrofuran-2-yl]-methyl [(2R,3S,4S)-3,4-dihydroxytetrahydrofuran-2-yl]-methyl dihydrogen diphosphate	Aldo-keto reductase (2WZM) [(2R,3R,4R,5R)-5-(6-amino-9H-purin-9-yl)- 3-hydroxy-4-(phosphonooxy)-tetrahydrofuran-2-yl]-methyl [(2R,3S,4S)-3,4-dihydroxytetrahydrofuran-2-yl]-methyl dihydrogen diphosphate (NA7)[9]	Aldose reductase (1AZ2) NADP nicotinamide-adenine-dinucleotide phosphate (NAP)[4]	Yvgn protein (3D3F) NADPH (NDP)[4]
3BLM	Beta-lactamase N-(1-carboxy-2-hydroxy-4-oxo-butyl)-N-(3-oxo-cispropenyl)-amine	Beta-lactamase (1GHP) Open form - penicillin g (PNM)[10]	Beta-lactamase (1BLC) N-(2-hydroxy-4-oxo-butyl)-N-(3-oxo-transpropenyl)amine (TEM)	Beta-lactamase (4XUZ) (3R,6S)-2-hydroxy-3-[(thiophen-2-ylacetyl)amino]-1,2-oxaborinan-6-ylacetic acid (4D6)[5]
3D0O	L-lactate dehydrogenase 1 Nicotinamide-adenine-dinucleotide	Lactate dehydrogenase (3D4P) Nicotinamide-adenine-dinucleotide (NAD)[4]	Lactate dehydrogenase (1LDN) Oxamic acid (OXM)[5]	Malate dehydrogenase (1UXJ) Nicotinamide-adenine-dinucleotide (NAD)[4]
3GSZ	RNA-directed RNA polymerase 2-(3-bromophenyl)-6-[(2-hydroxymethyl)-amino]-1H-benzo[de]isoquinoline-1,3(2H)-dione	RNA polymerase (3BR9) (2r)-2-(3-[5-hydroxy-2-(3-methylbutyl)-3-oxo-6-thiophen-2-yl-2,3-dihydropyridazin-4-yl]-1,1-dioxido-2h-1,2,4-benzothiadiazin-7-yloxy)propanamide (DEY)[5]	RNA polymerase (1YVX) 3-[isopropyl(4-methylbenzoyl)amino]-5-phenylthiophene-2-carboxylic acid (IPC)[5]	RNA-directed RNA polymerase (3CSO) (11S)-10-acetyl-11-[4-(benzyloxy)-3-chlorophenyl]-3,3-dimethyl-2,3,4,5,10,11-hexahydro-1h-dibenzo[b,e][1,4]diazepin-1-one (XNI)[5]
3I0C	ABO glycosyltransferase D-galactose	Fucosylgalactoside alpha N-acetylgalactosaminyltransferase (3V0N) 5-(5-formylthiophen-2-yl)uridine-5'(2-(acetylamino)-2-deoxy-alpha-D-galactosyl)-diphosphate (3GW)[5]	Galactosyltransferase (1ZIZ) Beta-D-galactose (GAL)[6]	Histo-blood group abo system transferase (5C1H) Octyl 3-deoxy-2-O-(6-deoxy-alpha-L-galactopyranosyl)-beta-D-xylo-hexopyranoside (DA8)[5]

Table 3: COACH's ligand/binding site recognition performance on the LigaSite non-redundant data-set containing 373 apo-proteins for which a corresponding holo structure is available (Desailly *et al.*, 2008). APO and HOLO Protein ID are the PDB codes of the corresponding three-dimensional structures. Ligand ID is the ligand name abbreviation used in the Protein Data Bank. Ligand type indicates the nature of the ligand: organic (O) or ion (I).

APO Protein ID	HOLO Protein ID	Ligand ID	Rank	Ligand details	Ligand type
180L	3GUM	PXY	1st	Substrate analog	O
1A4U	2BD0	NAP	1st	Coafactor analog	O
1ADE	1GIN	GDP	1st	Cofactor	O
1AK1	2HRE	PP9	1st	Substrate	O
1AKZ	3EUG	GOL	1st	Natural ligand	O
1ARB	3E0N	ARM	1st	Specific inhibitor	O
1ARL	3WC7	EF1	1st	Specific inhibitor	O
1B8E	1GX9	REA	1st	Specific ligand	O
1B8P	4PLT	NAI	1st	Cofactor	O
1BD9	1B7A	OPE	1st	Natural ligand analog	O
1BK7	1IYB	5GP	1st	Reaction product	O
1BKZ	4BLJ	70B	1st	Natural ligand analog	O
1BQC	5D9M	GLC	1st	Substrate	O
1BYI	4WOP	CTP	1st	substrate	O
1C48	1CQF	GLA	1st	Natural ligand	O
1C5H	1H4G	FXP	1st	Specific inhibitor	O
1CEO	1CEN	BGC	1st	Substrate analog	O
1CEX	1XZM	DPE	1st	Covalent inhibitor	O
1CPJ	2XU1	424	1st	Specific inibitor	O
1CRW	4WNC	NAD	1st	Cofactor	O
1CWY	1ESW	ACR	1st	Specific inhibitor	O
1DCO	1DCP	HBI	1st	Cofactor analog	O
1DHN	2NM2	NEU	1st	Substrate analog	O
1DQ0	3U4X	XMM	1st	Natural ligand analog	O
1DUP	1SEH	UMP	1st	Reaction product	O
1E4F	4PL7	ATP	1st	Substrate	O
1E5L	3ABI	NAD	1st	Cofactor	O
1E8Y	3QK0	QK0	1st	Specific inhibitor	O
1EDQ	3WL1	NAG	1st	Reaction product	O
1EPA	1EPB	REA	1st	Natural ligand	O
1EWZ	3FZC	MXF	1st	Inhibitor	O
1EY0	1A2T	THP	1st	Specific inhibitor	O
1EZI	3K8D	CTP	1st	Substrate	O
1F14	4DYD	NAD	1st	Cofactor	O
1F1S	1OJM	GCD	1st	Reaction product	O
1F2V	1I1H	COJ	1st	Reaction product	O
1F41	4IK7	IMN	1st	Specific ligand	O
1F5Z	4OE7	GXP	1st	Product analog	O
1FCQ	1FCV	NAG	1st	Substrate fragment	O
1FGB	1EEI	GAA	1st	Specific inhibitor	O
1FO9	2AM3	UPG	1st	Substrate analog	O
1FSF	1HOR	AGP	1st	Specific inhibitor	O
1FTF	2WDS	COA	1st	Substrate	O
1FTR	2FHJ	MFN	1st	Cofactor	O
1FWL	1H73	ANP	1st	Substrate analog	O

1G40	1Y8E	SVR	1st	Specific inhibitor	O
1G4E	1G4T	FTP	1st	Reaction product	O
1G95	1HM9	UD1	1st	Reaction product	O
1GBS	4CFP	AMV	1st	Substrate analog	O
1GCE	3O88	BSH	1st	Specific inhibitor	O
1GFS	3ICP	NAD	1st	Cofactor	O
1GOU	1GOY	3GP	1st	Substrate analog	O
1GSH	1GSA	ADP	1st	Reaction product	O
1GWK	1GWL	BMA	1st	Natural ligand	O
1GY0	1OG1	TAD	1st	Substrate analog	O
1HK9	4Y91	NUC	1st	Natural ligand	O
1HKA	1RAO	HH2	1st	Product analog	O
1HM5	3Q7I	6GP	1st	Reaction product	O
1HNK	1HND	COA	1st	Reaction product	O
1HO1	1M5W	DXP	3rd	Substrate	O
1I7N	1I7L	ATP	1st	Endogenous ligand	O
1IAD	1QJJ	Peptide	1st	Inhibitor	O
1ILV	2V4O	PO4	1st	Reaction product	I
1INL	2O0L	S4M	1st	Substrate analog	O
1J85	4PZK	SAH	1st	Cofactor	O
1J8S	1J8R	GAL	1st	Natural ligand	O
1JCF	4PKG	ATP	1st	Substrate	O
1JKS	3NAY	MP6	1st	Inibitor	O
1JXO	3WP0	Peptide	1st	Specific ligand	O
1JYK	4ZDQ	CTP	2nd	Substrate	O
1K0M	1EV9	GST	1st	Substrate analog	O
1K3O	3VPQ	GSH	1st	Substrate	O
1K6A	5D4Y	XYP	1st	Substrate	O
1KAM	1K4M	NAD	1st	Substrate	O
1KF5	IJVU	C2P	1st	Reaction product	O
1KHD	3R88	PRP	1st	Substrate	O
1KN9	1B12	1PN	1st	Specific inhibitor	O
1KPA	1RZY	5AS	1st	Substrate analog	O
1KWB	1EIR	BPY	1st	Substrate	O
1L7D	1L7E	NAI	1st	Reaction product	O
1LBV	1NV6	PI	1st	Reaction product	I
1LCI	2D1S	SLU	1st	Reaction intermediate analog	O
1LF4	3APR	Peptide	1st	Inhibitor	O
1LTU	1KW0	BH4	1st	Cofactor	O
1M1Z	1JGT	APC	1st	Substrate analog	O
1MKB	4KEH	1R3	1st	Substrate analog	O
1MMI	1D1E	Peptide	1st	Specific inhibitor	O
1MR7	3NZ2	ACO	1st	Substrate	O
1MTZ	1XQX	PCS	2nd	Specific inhibitor	O
1MWK	3KVG	ANP	1st	Substrate analog	O
1MZL	1UVC	STE	1st	Natural ligand	O
1N05	1N07	FMN	1st	Reaction product	O
1NDB	1S5O	152	1st	Substrate	O
1NON	4P83	U5P	1st	Specific ligand	O
1NXM	2IXC	TRH	1st	Substrate analog	O
1O24	3HZG	FAD	1st	Cofactor	O
1OEM	2CNH	IZB	1st	Specific inhibitor	O

1OFP	4UMB	0V5	1st	Specific inhibitor	O
1OGH	3TPS	DUP	1st	Substrate analog	O
1OGL	1CJE	DUN	1st	Substrate analog	O
1OGM	1OGO	GLC	1st	Reaction product	O
1OJQ	1GZF	NAD	1st	Cofactor	O
1OLZ	4GZ8	CA	1st	Specific ligand	I
1OOI	2GTE	VA	1st	Natural ligand	O
1OPY	2INX	FFP	2nd	Transition state analog	O
1OXT	1OXU	ADP	1st	Reaction product	O
1P1X	1UB3	HPD	1st	Reaction product	O
1P5H	2GCE	SFC	1st	Substrate analog	O
1P74	3PGJ	SKM	1st	Substrate	O
1PDB	3CL9	MTX	1st	Inhibitor	O
1PNG	1PNF	NDG	1st	Substrate analog	O
1Q52	4I42	1HA	1st	Product analog	O
1Q7M	2YCK	THG	1st	Substrate	O
1QCZ	2ATE	NIA	1st	Substrate analog	O
1QID	5EHN	5NZ	1st	Specific inhibitor	O
1QTO	5CJB	52G	1st	Specific ligand	O
1QTR	1XRR	Pro	10th	Substrate	O
1R0M	1SJC	SMG	2nd	Substrate	O
1R12	4F46	DVN	1st	Substrate analog	O
1R29	1R2B	Peptide	1st	Natural ligand	O
1RI5	1RI4	SAM	1st	Substrate	O
1RIQ	2ZZG	A5A	1st	Substrate analog	O
1RJ1	3OIB	IOD	2nd	Non-specific ligand	I
1RKM	1B0H	Peptide	1st	Specific ligand	O
1RZV	3GUH	ASO	1st	Substrate analog	O
1S2L	1S2G	3D1	1st	Substrate	O
1S7K	4LXY	ACO	1st	Substrate	O
1SGK	1DDT	APU	1st	Substrate analog	O
1SGZ	2FDP	FRP	1st	Inhibitor	O
1SJY	2A8P	MN	1st	Cofactor	I
1SNT	1F25	DAN	1st	Specific inhibitor	O
1SQG	3M6V	SAM	1st	COFACTOR	O
1SUL	1Z22	GDP	1st	Specific ligand	O
1SWH	4CPH	LH4	1st	Specific ligand analog	O
1TGN	4Z6A	0Z6	1st	Specific inhibitor	O
1THV	4TVT	ASC	3rd	Natural ligand	I
1TIB	1GT6	OLA	1st	Reaction product	O
1TJE	1TKE	SER	1st	Substrate analog	O
1TJV	2E9F	ARG	1st	Reaction product	O
1TVN	3NDZ	CT3	1st	Substrate analog	O
1TW0	1TXC	2AN	1st	Non-specific ligand	O
1TYF	4U0G	ZIL	1st	Substrate analog	O
1TYV	1TYU	TYV	2nd	Natural ligand	O
1TZV	3R2C	NUC	1st	Natural ligand	O
1U0T	1Z0U	NAP	1st	Reaction product	O
1U7U	1U7W	CTP	1st	Substrate	O
1UAJ	4YVK	SFG	1st	Substrate analog	O
1UBI	2D3G	Peptide	1st	Specific ligand	O

1UF4	1UF5	CDT	1st	Substrate	O
1ULU	1VL8	NAP	1st	Cofactor	O
1UMF	1QXO	FMN	1st	Cofactor	O
1UXZ	1UYX	BGC	1st	Specific ligand	O
1UYL	4YKQ	4EO	1st	Inhibitor	O
1V0S	1V0R	WO5	1st	Inhibitor	I
1V6Z	2CX8	SAH	1st	Cofactor	O
1V77				**No significant results**	
1VFJ	2XUL	ATP	1st	Specific ligand	O
1VIC	2XWL	CTP	1st	Substrate	O
1VJU	3DWR	0PA	1st	Substrate analog	O
1VPN	3BWR	SIA	1st	Specific ligand	O
1W9A	2AQ6	PLP	1st	Reaction product	O
1WJG	3HGM	ATP	1st	Specific ligand	O
1WNY	1WK8	VMS	1st	Substrate analog	O
1WOS	1WOO	THG	1st	Substrate	O
1WS9	2JIF	FAD	1st	Cofactor	O
1WTJ	1V9N	NDP	1st	Cofactor	O
1WXF	2PZ8	APC	1st	Substrate analog	O
1X56	3A5Y	KAA	1st	Substrate analog	O
1X7O	1X7P	SAM	1st	Cofactor	O
1X8F	3UND	PEP	1st	Substrate	O
1XIX	1P4N	Peptide	1st	Substrate	O
1XK7	1T3Z	CAO	1st	Substrate analog	O
1XO6	3TV5	RCP	1st	Inhibitor	O
1XQO	1XQP	8HG	1st	Substrate analog	O
1XW2	1WU5	XYP	2nd	Reaction product	O
1Y2Q	4RR6	A3S	1st	Substrate analog	O
1Y2T	4Z2S	NDG	1st	Endogenous ligand analog	O
1YBT	4WP8	ZDA	1st	inhibitor	O
1YVY	2GMV	PEP	1st	Reaction product	O
1Z7G	1HMP	5GP	1st	Reaction product	O
1ZAH	3TU9	5MM	1st	Specific inhibitor	O
1ZCU	3T7O	UPG	1st	Substrate	O
1ZNW	1ZNX	5GP	1st	Substrate	O
1ZTY	1ZU0	CBS	7th	Natural ligand	O
1ZUH	2IYR	SKM	2nd	Substrate	O
2A6Z	1N47	TNR	1st	Specific ligand	O
2A8F	2AIB	ERG	1st	Specific ligand	O
2AD1	1LS6	A3P	1st	Reaction product	O
2AHF	2AHG	UCD	1st	Substrate	O
2AHU	2AHV	COA	1st	Cofactor	O
2AMJ	4FGJ	FAD	1st	Cofactor	O
2B0J	3F46	I2C	1st	Cofactor	O
2B6P	2EVU	GOL	1st	Non-specific ligand	O
2B78	3VSE	SAH	1st	Cofactor	O
2B98	2C9D	PHR	1st	Inhibitor	O
2BGT	1IXY	UDP	1st	Reaction product	O
2BJW	4LZZ	08T	1st	Substrate analog	O
2BOE	2BOG	SGC	1st	inhibitor	O
2C61	3OAA	ANP	1st	Reaction product analog	O
2C7I	3A7R	LAQ	1st	Reaction intermediate	O

2CAR	3S86	IMP	1st	Reaction product	O
2CHS	1COM	PRE	1st	Reaction product	O
2CI3	1LXY	CYR	1st	Reaction product	O
2CWK	1F3F	D4T	1st	Substrate analog	O
2CX5	2Z0X	5CA	1st	Substrate analog	O
2D59	4XYL	COA	1st	Substrate	O
2DHQ	2BT4	CA2	1st	Specific inhibitor	O
2DJ6	3QNA	BIO	1st	Substrate	O
2DKA	2DKD	PO4	1st	Substrate	I
2DPS	2Z3O	PHE	1st	Substrate	O
2DQW	3TZF	HH2	1st	Substrate analog	O
2E0C	4AJ3	NAP	1st	Substrate	O
2E0K	2E0N	SAH	1st	Cofactor	O
2E10	2DTI	BT5	1st	Reaction intermediate	O
2E1V	2E1T	MLC	1st	Substrate	O
2E3S	2E3O	16C	1st	Substrate	O
2ECR	2R6V	FMN	1st	Substrate	O
2EX0	2ZWI	C5P	1st	Substrate analog	O
2F82	5HWQ	CAA	1st	Substrate	O
2F9T	5B8H	PAU	1st	Substrate	O
2FK7	1KPG	SAH	1st	Cofactor	O
2FP8	2V91	S55	2nd	Substrate	O
2FSF	2FSH	ANP	1st	Substrate analog	O
2G67	2QTC	TDK	1st	Reaction intermediate analog	O
2G95	2H3D	NMN	1st	Reaction product	O
2GFV	2BYZ	DAO	1st	Substrate analog	O
2GG4	2GGA	S3P	1st	Substrate	O
2GQV	2P4T	NAP	1st	Cofactor	O
2GSF	4HYH	1AM	1st	Specific inhibitor	O
2GT2	2GT4	GDD	1st	Substrate	O
2GUB	1S5N	XYL	1st	Inhibitor	O
2GWX	3VI8	13M	4th	Natural ligand analog	O
2GYY	1DSS	NAD	1st	Cofactor	O
2H2Z	2A5I	AZP	1st	Inhibitor	O
2HBJ	1ZBH	MG	1st	Cofactor	I
2HIV	2HIX	ATP	1st	Substrate	O
2HK0	2HK1	FUD	1st	Substrate	O
2HVM	1FFR	NAG	1st	Substrate	O
2HY7	2Q6V	UDP	1st	Substrate analog/reaction product	O
2HZR	2HZQ	STR	1st	Natural ligand	O
2I4L	2J3L	P5A	1st	Reaction intermediate analog	O
2IOB	2IO9	GSH	1st	Substrate	O
2IRU	3PKY	UTP	1st	Substrate	O
2J71	2J72	GLC	1st	Natural ligand	O
2J8N	2CY2	ACO	1st	Cofactor	O
2JBR	3PFD	FDA	1st	Cofactor	O
2NWD	1BB5	CTO	1st	Inhibitor	O
2NXC	3CJT	SAM	1st	Cofactor	O
2O9P	1OIM	NOJ	1st	Inhibitor	O
2OAM	3E1T	FAD	1st	Cofactor	O
2OPT	3B6A	ZCT	1st	Specific inhibitor	O
2OU1	1L6L	BOG	10th	Natural ligand analog	O

2OVE	2OVD	DAO	1st	Specific ligand	O
2PAW	4ZZZ	FSU	1st	Specific inhibitor	O
2PFK	2F48	FBP	1st	Reaction product	O
2PKF	4O1G	AGS	1st	Substrate analog	O
2PPN	1BL4	AP1	1st	Specific ligand	O
2Q5R	2JG1	ANP	1st	Cofactor analog	O
2Q6Z	1R3Q	1CP	1st	Reaction product	O
2QBV	5CKX	TSA	1st	Transition state analog	O
2QDK	1RXU	THM	1st	Substrate analog	O
2QEV	2QEH	SRO	1st	Specific ligand	O
2QSU	3DP9	BIG	1st	Substrate analog	O
2QVL	2QV7	ADP	1st	Reaction product	O
2QYS	3C1O	NAP	1st	Cofactor	O
2R60	3S27	FRU	1st	Substrate	O
2RG7	2R7A	HEM	1st	Specific ligand	O
2RJD	3H6Z	Peptide	3rd	Specific ligand	O
2SGA	3NCL	CCZ	1st	Specific inhibitor	O
2SIL	2AH2	FSI	1st	Inhibitor	O
2TS1	3TZL	TRP	1st	Substrate analog	O
2UYO	1RJD	SAM	1st	Cofactor	O
2V78	3QAI	XNN	1st	Inhibitor	O
2VFB	4B55	P18	1st	Specific inhibitor	O
2VFY	2VFK	AMP	1st	Specific ligand	O
2VQ4	2V8L	GLC	1st	Natural ligand	O
2VUA	3RSJ	GAL	1st	Specific ligand	O
2WN4	1GIR	NDP	1st	Substrate	O
2WVH	2VBI	TPP	1st	Cofactor	O
2WZT	3D3F	NDP	1st	Cofactor	O
2X5S	2PA4	UPG	1st	Product analog	O
2YWB	2YWB	AMP	1st	Product analog	O
2YXF	4RA3	TFX	2nd	Non-specific ligand	O
2YYA	3RV3	ADP	1st	Reaction product	O
2YYT	4MUZ	BMP	1st	Specific inhibitor	O
2YZG	1EZ1	ANP	1st	Substrate analog	O
2ZBS	5DKS	5C6	1st	Specific ligand	O
2ZCG	4MUZ	BMP	1st	Specific inhibitor	O
2ZCO	3WEH	PS7	1st	Reaction intermediate	O
2ZGL	2R0H	CTO	1st	Natural ligand	O
2ZHY	3KE5	ATP	1st	Substrate	O
2ZJ8	4F93	ATP	1st	Substrate	O
2ZTY	3ZZD	G85	1st	Specific inhibitor	O
3A0Y	3TZ5	ADP	1st	Reaction product	O
3A5Q	2D52	COA	1st	Substrate	O
3AAP	3CJA	ANP	1st	Specific inhibitor	O
3ADO	4OM8	NAD	1st	Cofactor	O
3APP	3K5C	0BI	1st	Specific inhibitor	O
3B3G	2FYT	SAH	1st	Cofactor	O
3BA1	4NJO	NAD	1st	Cofactor analog	O
3BLM	4XUZ	4D6	1st	inhibitor	O
3BTV	3NTQ	NAD	1st	Specific ligand	O
3BUE	2ZFZ	ARG	1st	Natural ligand	O
3BYL	1W2T	SUC	1st	Substrate	O

3C2E	1QPQ	NTM	1st	Substrate	O
3C8N	3B4Y	F42	1st	Cofactor	O
3CAF	3CU1	SCR	2nd	Specific ligand	O
3CB6				**No significant results**	
3CGZ	4PL9	ADP	1st	Reaction product	O
3COU	2A8S	GTP	2nd	Substrate analog	O
3CQ1	3CQ3	MG	1st	Non-specific ligand	I
3CRM	2ZE7	DST	2nd	Substrate analog	O
3CSR	3CT5	NAG	1st	Substrate analog	O
3CTB	4OK1	198	1st	Specific agonist	O
3D0O	1UXJ	NAD	1st	Cofactor	O
3D95	1FE3	OLA	1st	Ligand analog	O
3DRD	4ATP	PLP	1st	Cofactor	O
3DRE	4V7N	ADP	1st	Reaction product	O
3DUL	5FHQ	SAM	1st	Cofactor	O
3E1S	4C30	ANP	1st	Substrate analog	O
3E5B	2HJP	PPR	1st	Substrate analog	O
3EIZ	2AU9	MG	1st	Specific ligand	I
3EK6	2J4L	UTP	1st	Specific inhibitor	O
3ETF	4I8P	NAD	1st	Cofactor	O
3EX9	3JUM	AOD	2nd	Reaction intermediate analog	O
3EXR	1XBZ	LX1	1st	Reaction product	O
3F1L	1VL8	NAP	1st	Cofactor	O
3F6F	2IMI	GSH	1st	Substrate	O
3FTD	4GC9	SAM	1st	Cofactor	O
3FV6	4QFG	AMP	1st	Natural ligand analog	O
3G1S	3VE7	BMP	1st	Specific inhibitor	O
3GBT	1BWF	GOL	1st	Substrate analog	O
3GD0	3GD9	BCG	1st	Substrate/product analog	O
3GLK	3OUZ	ADP	1st	Reaction product	O
3GPG	3GQO	APR	1st	Specific ligand	O
3GQH	3SUC	ATP	1st	Specific ligand	O
3GSZ	3CSO	XNI	1st	Inhibitor	O
3H2G	2XLC	DEP	1st	Specific inhibitor	O
3H38	1MIY	CTP	1st	Substrate	O
3H49	3QAI	XNN	1st	Specific inhibitor	O
3H71	2YA7	ZMR	1st	Specific inhibitor	O
3HBH	3HBD	MXE	1st	Substrate mimic	O
3HIS	1MRG	ADN	1st	Substrate analog	O
3HJ4	2Q0D	ATP	1st	Substrate analog	O
3HNX	3HP8	SUC	1st	Specific ligand	O
3I0C	5C1H	DA8	1st	Inhibitor	O
3I3I	1KQU	BR4	1st	Substrate analog	O
3I8S	4R98	GNH	1st	Substrate analog	O
3IK8	3IKD	J9Z	1st	Specific inhibitor	O
3ILY	3ROF	Peptide	8th	Substrate mimic	O
3ITY	3ITL	LRH	1st	Reaction product	O
3IUJ	1O6F	PRO	1st	Substrate analog	O
3JYL	1H2B	NAJ	1st	Cofactor	O
3K0M	2POY	Peptide	1st	Specific ligand	O
3KAJ	3KAL	HGS	1st	Raction product	O
3KDH	3NMV	PYV	1st	Natural ligand analog	O

3KJE	1G3R	ACP	1st	Substrate analog	O
3KJT	5HZ7	MLR	1st	Natural ligand analog	O
3KP7	4EJW	SRY	1st	Specific inhibitor	O
3KR9	3KU1	SAM	1st	Cofactor	O
3KX7	3KVU	ACO	2nd	Substrate analog	O
3LIG	3PIJ	FRU	1st	Substrate mimic	O
3LOI	3LZZ	GDP	2nd	Specific ligand	O
3LXZ	4IS0	GDS	1st	Substrate analog	O
3M4D	3M4E	BCD	2nd	Natural ligand	O
3N6J	1ECQ	MG	1st	Specific ligand	I
3NK6	3NK7	SAM	1st	Cofactor	O
3PTE	3O88	BSH	1st	Specific inhibitor	O
3SSW	1JHG	TRP	1st	Specific ligand	O
3VZT	3A2S	SUC	4th	Specific ligand	O
4AKE	4EDH	ADP	1st	Reaction Product	O
4PGM	2IKQ	PO4	1st	Reaction product	O
4PTI	3LDJ	SCR	2nd	Specific ligand analog	O

Table 4: Table 4: LIBRA-WA's ligand/binding site recognition performance excluding from the ligand binding sites database the holo proteins present in the LigaSite non-redundant dataset (Dessailly *et al.*, 2008). APO and HOLO Protein ID are the PDB codes of the corresponding three-dimensional structures. Ligand ID is the ligand name abbreviation used in the Protein Data Bank.

APO Protein ID	HOLO Protein ID	Ligand ID	Rank	Ligand details
180L	186L	N4B	1st	Specific ligand analog
1A4U	1MG5	NAI	1st	Cofactor
1ADE	2V40	GDP	1st	Substrate
1AK1	1C9E	MP1	1st	Substrate analog
1AKZ	2C53	DUR	5th	Substrate analog
1ARB	4NSV	2OY	1st	Specific inhibitor
1ARL	3FVL	BHK	1st	Specific inhibitor
1B8E	3NQ3	DKA	2nd	Substrate analog
1B8P	1BMD	NAD	1st	Cofactor
1BD9	2B7A	OPE	1st	Natural ligand analog
1BK7	1UCA	U2P	1st	Reaction product
1BKZ	4UW6	VV7	1st	Natural ligand
1BQC	2MAN	MAN	1st	Reaction product
1BYI	3QXS	NAP	3rd	Substrate analog
1C48	1BOS	GLA	1st	Natural ligand
1C5H	2B46	XYP	1st	Reaction product
1CEO	3AOF	BMA	1st	Substrate
1CEX	1XZL	HEE	1st	Covalent inhibitor
1CPJ	2DC7	042	1st	Covalent inibitor
1CRW	2B4R	NAD	1st	Cofactor
1CWY	2OWC	ACR	1st	Specific inhibitor
1DCO	2V6T	H2B	3rd	Substrate-like ligand
1DHN	1RRY	204	1st	Inhibitor
1DQ0	4P9W	R3M	1st	Specific ligand analog
1DUP	1SYL	DUT	1st	Substrate analog
1E4F	4A2B	AGS	1st	Substrate analog

1E5L	2QG4	NAD	135th	Cofactor
1E8Y	3L13	JZW	1st	Specific inhibitor
1EDQ	1X6N	AO3	1st	Specific inhibitor
1EPA	2RA6	ETY	1st	Ligand analog
1EWZ	2WGI	PNM	1st	Specific inhibitor
1EY0	1A2T	THP	1st	Specific inhibitor
1EZI	1QWJ	NCC	1st	Reaction product
1F14	1F0Y	NAI	1st	Cofactor
1F1S	2BRP	SIE	1st	Specific inhibitor
1F2V	4AU1	P8X	1st	Reaction intermediate
1F41	1DVS	STL	1st	Specific inhibitor
1F5Z	4IMF	SI3	1st	Substrate
1FCQ	2ATM	MES	1st	Substrate analog
1FGB	1JQY	A32	1st	Specific inhibitor
1FO9	2APC	UDM	1st	Substrate analog
1FSF	1NE7	AGP	1st	Specific inhibitor
1FTF	3HYK	A3P	1st	Reaction product
1FTR				**No significant results**
1FWL	3BY5	NAI	2nd	Substrate analog
1G40	1Y8E	SVR	9th	Competitive inhibitor
1G4E	1G4S	TPS	1st	Reaction product
1G95	2W0W	LZS	1st	Inhibitor
1GBS	154L	NAG	2nd	Substrate analog
1GCE	1LLB	PCN	1st	Inhibitor
1GFS	1E6U	NAP	1st	Reaction product
1GOU	1GMP	2GP	1st	Substrate analog
1GSH	3R5X	ATP	1st	Cofactor
1GWK	1W8U	BMA	1st	Natural ligand
1GY0	1OG4	NAI	1st	Cofactor
1HK9	3QO3	ATP	1st	Natural ligand
1HKA	3ILJ	APC	2nd	Inhibitor
1HM5	4QFH	G6P	1st	Reaction product
1HNK	4NHD	COEA	1st	Product
1HO1	3F4N	PXP	1st	Reaction product
1I7N	1AUX	AGS	1st	Ligand analog
1IAD	1QJJ	HOA	1st	Inhibitor
1ILV	2E6B	WO4	5th	Specific inhibitor
1INL	2O0L	S4M	1st	Substrate analog
1J85	4JAL	SAH	1st	Cofactor
1J8S				**No significant results**
1JCF	4CZJ	ANP	1st	Substrate analog
1JKS	3ZXT	ACP	1st	Substrate analog
1JXO				**No significant results**
1JYK	2O63	UDP	20th	Substrate analog
1K0M	4K0N	MAL	38th	Natural ligand mimic
1K3O	4HJ2	LZ6	1st	Substrate analog
1K6A	1B30	XYP	1st	Reaction product
1KAM	1YUM	NCN	1st	Cofactor
1KF5	1R5C	CPA	1st	Substrate analog
1KHD	1ZXY	PRP	1st	Substrate
1KN9	1B12	1PN	1st	Specific inhibitor

1KPA	3RHN	5GP	1st	Substrate
1KWB	1EIR	BPY	1st	Substrate
1L7D	1NM5	NAD	1st	Substrate
1LBV	1LBY	F6P	1st	Product
1LCI	1BA3	MBR	1st	Inhibitor
1LF4	1LF2	R37	1st	Inhibitor
1LTU	1DMW	HBI	1st	Cofactor
1M1Z	3SEZ	ATP	1st	Substrate
1MKB	4KEH	1r3	1st	Substrate analog
1MMI	3QSB	743	1st	Specific inhibitor
1MR7	4HUR	ACO	1st	Substrate
1MTZ	1XRQ	LEU	1st	Substrate
1MWK	4A61	ANP	1st	Substrate analog
1MZL	1UVC	STE	1st	Natural ligand
1N05	1NB9	RBF	1st	Substrate
1NDB	2H3P	ACO	1st	Substrate
1NON	4P86	5GP	1st	Substrate analog
1NXM	1DZT	ATY	1st	Substrate analog
1O24	4KAT	FDA	1st	Cofactor
1OEM	2QBP	527	1st	Inhibitor
1OFP	1OF6	DTY	1st	Specific inhibitor
1OGH	3TPS	DUP	4th	Substrate analog
1OGL	2YB0	DUR	1st	Substrate analog
1OGM	3WWG	BGC	1st	Substrate analog
1OJQ				**No significant results**
1OLZ				**No significant results**
1OOI	3B88	ETE	1st	Substrate analog
1OPY	3OWY	EQU	1st	Transition state analog
1OXT	3C4J	AGS	1st	Nucleotide analogs
1P1X	1JCJ	HPD	1st	Reaction intermediate
1P5H	1T3Z	CAO	1st	Substrate analog
1P74	3PHJ	DHK	1st	Reaction product
1PDB	1BOZ	NDP	1st	Cofactor
1PNG	3PMS	GOL	1st	Non-specific ligand
1Q52	4QII	2NE	1st	Substrate analog
1Q7M	2E7F	2CF	1st	Substrate analog
1QCZ	2FWP	ICR	1st	Substrate
1QID	1H23	E12	1st	Specific inhibitor
1QTO	4IAG	GOL	1st	Non-specific ligand
1QTR	1MU0	PHK	22nd	Inhibitor
1R0M	1SJB	OSB	1st	Product
1R12	3ZWP	AVU	1st	Substrate analog
1R29	4CP3	RBT	1st	Specific inhibitor
1RI5	3BGV	SAH	1st	Reaction product
1RIQ	3HXQ	A5A	1st	Substrate analog
1RJ1	2CJ7	IOD	1st	Non-specific ligand
1RKM				**No significant results**
1RZV	3CX4	GLC	1st	Substrate
1S2L	1S2D	AR4	1st	Substrate analog
1S7K				**No significant results**
1SGK	1DTP	APU	1st	Substrate analog
1SGZ	3I25	MV7	1st	Specific inhibitor

1SJY	1SOI	SM	1st	Non-specific ligand
1SNT	2F25	DAN	1st	Inhibitor
1SQG	4FP9	SAM	1st	Cofactor
1SUL				**No significant results**
1SWH	3WZP	ZOF	1st	Specific ligand analog
1TGN	1C2F	BAH	1st	Specific inhibitor
1THV	4TVT	NA	2nd	Non-specific ligand
1TIB	1GT6	OLA	1st	Reaction product
1TJE	1TKE	SER	1st	Substrate analog
1TJV	1DCN	AS1	1st	Substrate analog
1TVN	4A3H	DCB	1st	Substrate analog
1TW0	2QIM	ZEA	3rd	Non-specific ligand
1TYF	1TG6	FME	113th	Reaction product
1TYV	3TH0	PZU	1st	Natural ligand analog
1TZV				**No significant results**
1U0T	4DY6	A22	1st	Substrate analog
1U7U				**No significant results**
1UAJ	1P9P	SAH	1st	Substrate analog
1UBI	4WZP	SEP	1st	Specific ligand
1UF4	1UF5	CDT	1st	Substrate
1ULU	2QIO	NAD	1st	Cofactor
1UMF				**No significant results**
1UXZ	1W9W	BGC	1st	Specific ligand
1UYL	3T0Z	ATP	1st	Substrate
1V0S	2ZE9	PD7	1st	Reaction product
1V6Z	3KW2	ADN	3rd	Substrate analog
1V77				**No significant results**
1VFJ	4CNZ	ADP	1st	Specific ligand
1VIC	1H7H	CDP	1st	Substrate analog
1VJU				**No significant results**
1VPN	1SIE	SIA	1st	Specific ligand
1W9A	4QVB	F42	1st	Cofactor
1WJG	3QTB	D5M	1st	Reaction product analog
1WNY	1WK8	VMS	1st	Substrate analog
1WOS	3AOI	C2F	1st	Reaction product analog
1WS9	2DVL	FAD	1st	Cofactor
1WTJ				**No significant results**
1WXF	1EE1	ATP	1st	Substrate analog
1X56	2XTI	NB8	1st	Substrate analog
1X7O	1ZJR	GOL	40th	Substrate analog
1X8F	2NXH	PEP	1st	Substrate
1XIX				**No significant results**
1XK7	1XA4	COA	1st	Substrate analog
1XO6	3IB9	BTN		Substrate
1XQO				**No significant results**
1XW2	2B4F	XYP	1st	Reaction product
1Y2Q	3PD2	A3S	1st	Substrate analog
1Y2T	2OFD	NGA	1st	Endogenous ligand
1YBT	1WC1	TAT	61st	Substrate analog
1YVY	1AQ2	ATP	1st	Substrate
1Z7G	4RAB	3L3	1st	Specific inhibitor
1ZAH	3MMT	2FP	1st	Substrate
1ZCU	1LL2	UPG	1st	Substrate

1ZNW	2F3T	LGP	1st	Substrate analog
1ZTY				**No significant results**
1ZUH	1ZUI	SKM	1st	Substrate
2A6Z				**No significant results**
2A8F	1LRI	CLR	1st	Specific ligand
2AD1	1BO6	VO4	2nd	Transition state analog
2AHF	2FUZ	MPD	1st	Non-specific ligand
2AHU				**No significant results**
2AMJ	3RPE	FAD	1st	Cofactor
2B0J	3F46	I2C	1st	Cofactor
2B6P	2B6O	MC3	1st	Specific ligand analog
2B78	3VSE	SAH	1st	Cofactor
2B98				**No significant results**
2BGT	1NZF	GOL	1st	Substrate analog
2BJW	2VII	AMP	1st	Substrate analog
2BOE	2BOD	BGC	1st	Reaction product
2C61	3B2Q	ATP	1st	Reaction product
2C7I	3R07	MPD	1st	Substrate analog
2CAR				**No significant results**
2CHS	3ZP7	ISJ	1st	Substrate
2CI3	2CI1	KOR	1st	Specific inhibitor
2CWK	2HVD	ADP	1st	Substrate
2CX5				**No significant results**
2D59	3Q9N	COA	1st	Natural ligand
2DHQ	3N76	CA2	1st	Specific inhibitor
2DJ6	1Y13	BIO	1st	Substrate
2DKA	3UW2	ZN	93rd	Inhibitor
2DPS	2Z3P	LEU	1st	Substrate
2DQW	1EYE	PMM	1st	Substrate analog
2E0C	1XKD	NAP	1st	Substrate
2E0K	1S4D	GOL	6th	Substrate analog
2E10	1WQW	BT5	1st	Substrate analog
2E1V	2XR7	MLC	5th	Substrate
2E3S	2Z9Z	DDR	1st	Substrate
2ECR	1RZ1	NAD	1st	Cofactor
2EX0	3S44	FN5	1st	Subtrate analog
2F82	2WYA	HMG	1st	Reaction product
2F9T	3BF3	PAZ	1st	Reaction product
2FK7	1KPI	SAH	1st	Cofactor
2FP8	2V91	S55	1st	Substrate
2FSF	3BXZ	ADP	1st	Reaction product
2G67	3AI7	TPP	1st	Reaction intermediate
2G95	4M6Q	20T	1st	Specific inhibitor
2GFV	4C70	TLJ	1st	Inhibitor
2GG4	1RF6	S3P	1st	Substrate
2GQV	2P4T	NAP	4th	Cofactor
2GSF	4P5Z	Q7M	1st	Specific inhibitor
2GT2	2GT4	GDD	1st	Substrate
2GUB	4QDW	LAI	1st	Reaction intermediate
2GWX	3GWX	EPA	1st	Natural ligand
2GYY	4R3N	NAP	1st	Cofactor
2H2Z	2A5K	AZP	1st	Inhibitor

2HBJ	2HBK	MN	1st	Cofactor
2HIV	4EQ5	AMP	1st	Reaction product
2HK0	3VNL	TAG	1st	Substrate
2HVM	1KQY	NAG	1st	Substrate
2HY7				**No significant results**
2HZR	40SD	PLM	1st	Natural ligand analog
2I4L	2J3L	P5A	1st	Substrate analog
2IOB	3A2Y	TS5	1st	Substrate
2IRU	2FAR	DTP	1st	Substrate analog
2J71				**No significant results**
2J8N	3DR8	ACO	1st	Cofactor
2JBR				**No significant results**
2NWD	4XAD	3ZW	1st	Substrate analog
2NXC	3CJT	SAM	1st	Cofactor
2O9P	2JIE	G2F	1st	Product analog
2OAM	2APG	FAD	1st	Cofactor
2OPT				**No significant results**
2OU1	4JGX	PLM	1st	Natural ligand
2OVE	1EPB	REA	1st	Natural ligand analog
2PAW	4HHY	15R	1st	Specific inhibitor
2PFK	4XYK	ADP	1st	Reaction product
2PKF	4O1G	AGS	1st	Substrate analog
2PPN	1BL4	AP1	1st	Specific ligand
2Q5R	2JG1	ANP	1st	Substrate analog
2Q6Z	1R3Q	ACP	1st	Reaction product
2QBV	2VKL	MLT	1st	Product analog
2QDK	1RXU	THM	1st	Reaction product analog
2QEV	2QEO	LNR	1st	Natural ligand
2QSU	2H8G	ADE	1st	Reaction product
2QVL	4WER	AMP	1st	Reaction product analog
2QYS	2QW8	NAP	1st	Cofactor
2R60	2R66	F6P	1st	Substrate
2RG7	1AIJ	BPH	55th	Endogenous ligand analog
2RJD	3P8H	P8H	1st	Specific inhibitor
2SGA	2QAA	TYR	1st	Reaction intermediate
2SIL	1N1T	DAN	1st	Specific inhibitor
2TS1	1VBM	YSA	1st	Substrate analog
2UYO				**No significant results**
2V78	2DCN	ADP	1st	Reaction product
2VFB	4NV7	COA	1st	Cofactor
2VFY	4YEZ	A2P	13th	Specific ligand analog
2VQ4	4BFN	GLC	2nd	Natural ligand
2VUA	3RSJ	GAL	7th	Natural ligand
2WN4	2WN6	NDP	1st	Cofactor
2WVH	3OE1	TDL	1st	Reaction intermediate
2WZT	1S2C	FLF	9th	Specific inhibitor
2X5S	2X65	M1P	1st	Substrate
2YWB	1GPM	AMP	1st	Product analog
2YXF	1LDS	NA	10th	Non-specific ligand
2YYA	2XD4	ADP	1st	Substrate analog
2YYT	2CZF	XMP	1st	Product analog

2YZG	4L1K	ANP	1st	Substrate analog
2ZBS	1S9Q	OHT	1st	Natural ligand analog
2ZCG	3MWA	UFT	1st	Covalent inhibitor
2ZCO	3ADZ	PS7	1st	Reaction intermediate
2ZGL	2ZGO	LAT	1st	Natural ligand analog
2ZHY	3KE5	ATP	1st	Substrate
2ZJ8	2I4I	AMP	2nd	Specific ligand
2ZTY	3ZZ9	G83	1st	Specific inhibitor
3A0Y	1Y8O	ADP	38th	Reaction product
3A5Q	1CHW	HXC	1st	Substrate analog
3AAP	4BR7	AU1	1st	Specific inhibitor
3ADO	3F3S	NAD	1st	Cofactor
3APP	1PPM	0P1	1st	Specific inhibitor
3B3G	2Y1W	SFG	1st	Cofactor analog
3BA1	2DBQ	NAP	1st	Cofactor
3BLM	1GHP	PNM	1st	Reaction intermediate
3BTV				**No significant results**
3BUE	2ZFZ	ARG	1st	Natural ligand
3BYL	1PT2	SUC	1st	Substrate
3C2E	3C2R	PHT	1st	Specific inhibitor
3C8N	4ICC	MLY	6th	Non-specific ligand
3CAF	1E0O	SGN	1st	Specific ligand
3CB6				**No significant results**
3CGZ	2GR7	C8E	1st	Specific inhibitor mimic
3COU	2A8S	GTP	1st	Substrate analog
3CQ1				**No significant results**
3CRM	3CRQ	DPO	1st	Reaction product
3CSR				**No significant results**
3CTB	1ILH	SRL	1st	Specific agonist
3D0O	1LDN	NAD	1st	Cofactor
3D95	1CBQ	RE9	1st	Natural ligand analog
3DRD	3DOD	PLP	1st	Cofactor
3DRE	3JU6	ANP	1st	Substrate analog
3DUL	1SUS	SAH	1st	Reaction product
3E1S	2EWV	ADP	11th	Substrate analog
3E5B	3P0X	PYR	1st	Reaction product analog
3EIZ	3EIY	POP	1st	Substrate
3EK6	2BND	UDP	1st	Reaction product
3ETF	4ITA	NDP	1st	Cofactor
3EX9	3FF0	GOL	1st	Substrate mimic
3EXR	1KW1	LG6	1st	Substrate analog
3F1L	3GY0	NAP	1st	Cofactor
3F6F	1PN9	GTX	1st	Specific inhibitor
3FTD	2ZBR	SFG	1st	Cofactor analog
3FV6	2QR1	ADP	1st	Natural ligand
3G1S	3G1F	2OM	1st	Substrate analog
3GBT	2W41	ADP	2nd	Reaction product
3GD0				**No significant results**
3GLK	3JRX	S1A	1st	Specific ligand
3GPG	3GQO	APR	1st	Specific ligand
3GQH				**No significant results**
3GSZ	3BR9	DEY	1st	Specific inhibitor

3H2G	1GKK	SEP	2nd	Specific ligand
3H38	4WC0	ATP	1st	Substrate
3H49	2FV7	ADP	1st	Reaction product
3H71	2YA6	DAN	1st	Specific inhibitor
3HBH	2DKV	MES	1st	Substrate mimic
3HIS	2GA4	ADE	1st	Reaction product
3HJ4	4FHY	3AT	1st	Substrate analog
3HNX	2YHH	MAN	1st	Natural ligand
3I0C	3V0N	3GW	1st	Specific inhibitor
3I3I	1QLL	TDA	1st	Specific inhibitor
3I8S	3WIC	GNP	1st	Substrate analog
3IK8	4TYO	39X	1st	Specific inhibitor
3ILY				**No significant results**
3ITY	3ITV	PSJ	1st	Substrate
3IUJ	4BCC	JKT	2nd	Covalent inhibitor
3JYL	1QOR	NDP	1st	Cofactor
3K0M	4J5A	67Z	1st	Specific inhibitor
3KAJ	2WYO	GSH	1st	Product analog
3KDH	3NJ0	PYV	1st	Natural ligand mimic
3KJE	1YR7	GSP	1st	Substrate analog
3KJT	4IKM	TYI	1st	Modified amino acid
3KP7	4EJW	SRY	1st	Specific inhibitor
3KR9	3DXY	SAM	84th	Substrate
3KX7	3KVU	ACO	1st	Cofactor
3LIG	3LFI	BGC	1st	Substrate
3LOI	1XE8	GUN	3rd	Specific ligand analog
3LXZ	3UBL	GSH	1st	Substrate
3M4D	3M3R	BCD	1st	Natural ligand
3N6J	1ECQ	DXG	1st	Substrate analog
3NK6	3GYQ	SAM	1st	Substrate
3PTE	1YQS	BSA	1st	Specific inhibitor
3SSW	1ZT9	TRP	1st	Specific ligand
3VZT	3VZU	ANP	1st	Specific ligand
4AKE	3LR3	ADP	1st	Reaction product
4PGM	1E59	VO3	1st	Specific inhibitor
4PTI	1YLC	ABA	1st	Modified amino acid

REFERENCES

Atzeni, P., Polticelli, F., Toti, D. (2011) A framework for semi-automatic identification, disambiguation and storage of protein-related abbreviations in scientific literature, *Proceedings - International Conference on Data Engineering*, **5767646**, 59-61

Atzeni, P., Polticelli, F., Toti, D. (2011) An automatic identification and resolution system for protein-related abbreviations in scientific papers, *Lecture Notes in Computer Science*, **6623**, 171-176

Brylinski, M., Skolnick, J. (2008) A threading-based method (FINDSITE) for ligand-binding site prediction and functional annotation. *Proc Natl Acad Sci U S A*, **105(1)**, 129-134

Capra, J.A., Laskowski, R.A., Thornton, J.M., Singh, M., Funkhouser, T.A. (2009) Predicting Protein Ligand Binding Sites by Combining Evolutionary Sequence Conservation and 3D Structure. *PLoS Comput Biol*, **5(12)**

Furnham, N., Holliday, G.L., de Beer, T.A., Jacobsen, J.O., Pearson, W.R., Thornton, J.M. (2014) The Catalytic Site Atlas 2, *Nucleic Acids Res.*, **42**, D485-D489

Haas, J., Roth, S., Arnold, K., Kiefer, F., Schmidt, T., Bordoli, L., Schwede, T. (2013) The Protein Model Portal–a comprehensive resource for protein structure and model information, *Database (Oxford)*, 2013:bat031.

Levy, et al., (2015) retrieved from the web, http://home.wlu.edu/ levys/software/kd/

Dessailly, B.H., et al. (2008) LigASite: a database of biologically relevant binding sites in proteins with known apo-structures, *Nucleic Acids Res*, **36**, 667-673

Laskowski, R.A., et al. (2005a) ProFunc: a server for predicting protein function from 3D structure, *Nucleic Acids Res.*, **3**, W89-W93

Laskowski, R.A., et al. (2005b) Protein function prediction using local 3D templates, *J Mol Biol*, **351**, 614-626

Roy, A., Yang, J., Zhang, Y. (2012) COFACTOR: an accurate comparative algorithm for structure-based protein function annotation. *Nucleic Acids Research*, **40**, W471-W477

Toti, D., Atzeni, P., Polticelli, F. (2012) Automatic Protein Abbreviations Discovery and Resolution from Full-Text Scientific Papers: The PRAISED Framework, *Bio-Algorithms and Med-Systems*, **8**, 13-

Viet Hung, L., Caprari, S., Bizai, M., Toti, D., Polticelli, F. (2015) LIBRA: LIgand Binding site Recognition Application, *Bioinformatics*, **31(24)**, 4020-4022

Yang, J., Roy, A., Zhang, Y. (2013) Protein-ligand binding site recognition using complementary binding-specific substructure comparison and sequence profile alignment, *Bioinformatics*, **29(20)**, 2588-95
